# Wheat forage quality: significance, genetic and genomic advances, knowledge gaps, and future directions

**DOI:** 10.3389/fpls.2026.1935720

**Published:** 2026-10-05

**Authors:** Maria Ali, Ehsan Shakiba, Ali M. Missaoui, Mohamed Mergoum

**Affiliations:** 1 Department of Crop and Soil Sciences, University of Georgia, Griffin, GA, United States; 2 Independent Researcher, Batesville, AR, United States

**Keywords:** cool-season forage, dual-purpose wheat, forage nutritive value, genome-wide association study, genomic selection, grazing management, near-infrared reflectance spectroscopy

## Abstract

Winter wheat is grown worldwide primarily for grain, yet it also serves as an important cool-season forage, supporting livestock production when other feed is scarce. Wheat can be utilized as grazed pasture or harvested as hay, silage, and straw, and is widely used as dual-purpose (forage + grain) and double-cropping systems. During vegetative growth, wheat forage is highly palatable and nutritive, typically containing 20-30% crude protein (CP), and >80% digestibility, supporting rapid liveweight gains in beef cattle. However, forage quality is highly dynamic: as wheat matures, CP and energy decline, while neutral detergent fiber (NDF), acid detergent fiber (ADF), and lignin increase, reducing intake potential and digestibility. Forage quality is also affected by moisture, fertilization, and wheat class. At comparable developmental stages, winter and spring wheat generally show little inherent differences in forage quality, indicating that maturity and management are the major determinants of quality. This review synthesizes current knowledge on wheat forage in dual-purpose and grazing systems, emphasizing dry matter, CP, NDF, ADF, acid detergent lignin, relative feed value, and relative forage quality as key indicators of biomass, nutritive value, intake potential, and digestibility. We further assess developmental shifts in these traits and their consequences on livestock intake, animal performance, and grazing management, together with forage evaluation methods such as near-infrared reflectance spectroscopy. We also examine genetic and genomic approaches for improving forage traits. Wheat breeding has historically prioritized grain yield and straw characteristics, genomic dissection of forage quality remains limited. Nevertheless, recent genome-wide association, QTL mapping, and genomic selection studies targeting water-soluble carbohydrates, fiber fractions, and digestibility-related traits highlight promising avenues for improvement. Overall, wheat forage can provide exceptionally valuable winter feed resources, but its quality is governed by strong developmental, environmental, and genetic effects. Improved screening methods and breeding targets, such as combining high protein and energy with agronomic resilience, are needed to exploit wheat’s dual-purpose potential. This review integrates agronomic, animal nutrition, and genetic perspectives to guide future research and breeding of high-quality forage wheat.

## 1 Introduction

Winter wheat (*Triticum aestivum* L.), including soft red winter wheat, is grown worldwide primarily for grain. Yet, it also functions as a strategically important cool-season forage that supports livestock production when seasonal forage availability is constrained (Ul Haq et al., 2021). Wheat forage can be utilized through direct grazing or harvested as hay or silage, and it is frequently incorporated into integrated crop-livestock systems, including double-cropping and mixed-enterprise production systems that diversify farm income through both grain and livestock outputs (Sartin et al., 2023). In the U.S., wheat pasture plays a particularly important role in mixed crop-livestock systems across the semi-arid Southern Great Plains, which includes Texas, Oklahoma, and parts of New Mexico and Colorado, where dual-purpose production (winter grazing followed by grain harvest) is a dominant regional practice (Gunter and Combs, 2019). As one example of the scale and economic importance of these systems, Oklahoma reports winter wheat as its largest cash crop by acreage and documents substantial economic value even before including the added value generated through stocker grazing (DeVuyst et al., 2021). The decision to manage wheat for grain, forage, or dual-purpose use depends on both market conditions and production environment. Grain-only production is generally favored when grain prices are strong, yield potential is high, winter forage demand is low, or when soil moisture, stand establishment, or grazing management conditions make livestock use risky. Forage-only or grazeout systems may be more attractive when beef prices are high relative to wheat prices, when winter feed is scarce, or when drought, freeze injury, disease, or poor stand conditions reduce the likelihood of profitable grain harvest. Dual-purpose wheat provides an intermediate strategy by generating livestock income from fall–winter grazing while retaining the option for grain harvest, but its success depends on adequate fall forage growth, proper stocking rate, sufficient soil fertility, timely removal of cattle before first hollow stem, and favorable recovery conditions after grazing. Therefore, whole-system profitability is determined not only by grain yield but also by livestock weight gain, feed replacement value, cattle and wheat market prices, management costs, and the magnitude of any grain-yield penalty after grazing. In regions such as the Southern Great Plains, where winter wheat can provide high-quality forage during periods of limited pasture availability, dual-purpose management can improve farm income diversification and resilience when grazing is carefully managed (Gunter and Combs, 2019; DeVuyst et al., 2021). Beyond the U.S., wheat forage and dual-purpose cereal production systems are also used across diverse agroecological environments, including Australia, Argentina, Canada, and multiple Mediterranean-adjacent regions (Assmann et al., 2014; Cheng et al., 2016; McCormick et al., 2021; Arzadun et al., 2006).

Wheat forage is widely recognized as a highly palatable and nutrient-dense fodder at the vegetative stage. It is typically characterized by high crude protein, substantial levels of digestible energy, and meaningful mineral content with comparatively low fiber properties that support high animal intake and performance when grazing is managed appropriately (Lollato et al., 2017). Cattle grazing winter wheat pastures can achieve substantial average daily gains depending on forage availability, stocking rate, and environmental conditions (Ball et al., 2001). Nevertheless, wheat forage quality is highly dependent on plant developmental stage and management practices. Crude protein and digestibility can decline sharply as plants progress from vegetative growth toward heading and reproductive stages, while fiber fractions increase reduces both forage quality and intake potential (Sadeghpour et al., 2022). Consequently, wheat forage quality should be considered a multidimensional concept encompassing not only chemical composition, but also functional characteristics such as digestibility, palatability, and potential anti-nutritional or animal health risks associated with grazing lush cereal pastures under certain conditions (Maulana et al., 2019b).

Despite the clear agronomic and livestock production benefits of wheat forage, targeted genetic and genomic research on wheat forage remains limited compared with the extensive genetics efforts devoted to grain yield and end-use grain quality (Maulana et al., 2019a). This gap is important because forage biomass, crude protein, fiber concentration, digestibility, and related quality traits can vary among wheat genotypes and may be improved through targeted breeding when adequate phenotypic and genetic information is available. Therefore, this review article synthesizes existing knowledge is needed to better understand the potential for genetic improvement of forage traits in wheat. This review provides an updated overview of wheat as a forage and dual-purpose crop, emphasizing its global significance, production systems, and utilization. It summarizes the major forage quality traits, their variation across developmental stages, and the environmental and management factors governing forage quality. The review further highlights recent advances in the genetics and genomics of wheat forage quality, with particular emphasis on quantitative trait loci (QTL), genome-wide association studies (GWAS), genomic selection (GS), and other molecular breeding strategies for improving forage quality and dual-purpose performance. Finally, it identifies key research gaps and outlines future opportunities for integrating genomic technologies into breeding programs aimed at developing superior dual-purpose wheat cultivars.

## 2 Variation in wheat forage quality among genotypes, growth habits, and production systems

Wheat market classes, such as hard red winter, soft red winter, hard red spring, soft white, hard white, and durum, are primarily defined by grain characteristics and end-use quality rather than by vegetative tissue composition or forage feeding value (Clark and Bayles, 1935). Therefore, market class alone is not an ideal framework for interpreting wheat forage quality. A more useful framework considers genotype, winter or spring growth habit, vernalization and photoperiod sensitivity, maturity stage, first-hollow-stem timing, canopy structure, leaf-to-stem ratio, and the intended production system (Ball et al., 2001). These factors influence how long plants remain vegetative and leafy, when stem elongation begins, how rapidly structural carbohydrates accumulate, and whether the crop is managed as pasture, hay, whole-crop silage, straw, or a dual-purpose crop. For example, winter wheat in the Great Plains is often grazed during vegetative growth, when crude protein concentration is high and fiber concentration is relatively low, whereas spring wheat used as greenfeed, hay, or silage is often harvested at later developmental stages, when fiber concentration has increased and digestibility has declined (Shroyer et al., 1990; Ball et al., 2001; Capstaff and Miller, 2018).

Genotypic differences are important because wheat cultivars vary in phenology, canopy architecture, biomass production, leaf-to-stem ratio, and the timing of developmental transitions that determine forage quality. Differences in vernalization requirement and photoperiod sensitivity influence the rate of spring development and the duration of the high-quality grazing window. In dual-purpose wheat systems, first hollow stem is a particularly important management threshold because grazing beyond this stage can reduce subsequent grain yield; therefore, cultivar differences in first-hollow-stem timing affect both forage-use opportunity and grain-yield protection (Lollato et al., 2017; Kim and Anderson, 2015; West et al., 2024). Thus, wheat forage quality should be evaluated within a genotype × growth stage × management framework rather than primarily by market class. One of the few class-associated differences reported in the literature relates to the potential risk of bloat during grazing. In a controlled grazing experiment, soft red winter wheat exhibited greater bloat potential than hard red winter wheat, as indicated by increased rumen-fluid foam strength and viscosity in ruminally cannulated heifers (Akins et al., 2009). However, the actual incidence of bloat among stocker cattle remained low and did not differ significantly between classes, highlighting the complex and multifactorial nature of bloat risk in wheat pasture systems (Akins et al., 2009).

## 3 Conceptual frameworks for wheat forage quality

Forage quality is best defined in functional terms as the capacity of a fodder to support a targeted level of animal performance (e.g., maintenance, growth, reproduction, or milk production) when it is consumed under a given management system. It includes not only how much of the forage the animals consume but also how well the nutrients in it are utilized to convert them into animal products (Fulgueira et al., 2007; Moore et al., 2020; Newman et al., 2009). In the wheat forage system, this distinction is very important because vegetative wheat may have high crude protein concentration and digestibility, while animal performance still depends on forage availability, grazing pressure, sward structure, maturing stage, and animal nutrient requirements. In this framing, forage quality is not synonymous with laboratory composition alone, rather it integrates (1) the nutritive value of the forage (nutrient concentration and potential digestibility) and (2) the voluntary intake achieved by the animal, because intake largely determines how much digestible nutrient and energy enter the animal body (Cherney and Parsons, 2020). Consequently, interpretation of wheat forage quality should explicitly distinguish between “what is in the forage” and “what the animal consumes and utilizes”. Forage nutritive value is the chemical composition of forage that includes its energy, crude protein, fiber, vitamins, minerals, and other necessary nutrients. It shows the potential of the forage to sustain the performance of animals and is dependent on chemical and biological characteristics. Conversely, the amount of forage an animal eats (usually on a daily basis) is called the forage intake, which is influenced by a variety of chemical (e.g., the fiber concentration) and physical (e.g., the bulk density, the texture) factors that affect the palatability of forages and the rumen fill (Riaz et al., 2014). It should be noted that not only the forage plant species or cultivar determines the forage quality but also the animal species and class. Different herbivores may have varying nutritional values of the same forage based on physiological needs, digestive capacity, and feeding behavior and thus affect its relative value in the various types of livestock (Van Soest, 1994).

A large body of forage science, summarized in foundational work by Mertens (1994), emphasizes that variation in animal performance is often explained more by differences in intake than by digestibility alone, particularly when forages constitute much of the diet. Differences in physiological state (e.g., lactation), gut capacity, selective grazing behavior, and nutrient requirements can change the realized value of a given forage, even when the forage’s laboratory profile is unchanged (Mertens, 1994). When the quality of the forage is insufficient, forage alone might not meet to sustain the nutritional needs required to reach desired levels of animal performance. In this case, there is a need to supplement the livestock diets with extra sources of proteins and energy to counter the nutritional shortfalls of the forage (Adesogan et al., 2002).

## 4 Near-infrared reflectance spectroscopy for wheat forage quality assessment

Because wheat forage quality varies with genotype, maturity stage, seasonal conditions, and management practices, laboratory testing is necessary to accurately characterize nutritive value. Although direct assessment of forage nutritive value using animal feeding trials is a reliable method, it is not always a viable procedure because it is quite expensive and time-consuming. Thus, indirect analytical approaches such as enzymatic digestion, wet chemistry analysis, and *in vitro* fiber digestibility are frequently used due to their relative accuracy (Siahsar et al., 2009). Nevertheless, they are labor-intensive when large sample numbers are involved. To overcome these limitations, forage scientists and animal nutritionists have adopted Near-Infrared Reflectance Spectroscopy (NIRS) as a rapid, high-throughput method for forage quality assessment (Hancock et al., 2014). In contrast to wet chemistry, NIRS has become a cornerstone method because it can rapidly predict multiple compositional traits and digestibility once robust calibrations models are established (Shenk and Westerhaus, 1994). NIRS is a non-destructive, computerized analytical method based on absorbance in the near-infrared region (commonly ~1100–2500 nm, depending on instrument configuration) to estimate chemical attributes via molecular bond vibrations (Beever and Mould, 2000). Importantly, NIRS does not directly measure crude protein, fiber, or digestibility; instead, it predicts these traits from spectral using calibration equations developed from reference laboratory data (Stuth et al., 2003; Shenk and Westerhaus, 1994). These relationships make it possible to accurately and quickly estimate components such as neutral and acid detergent fiber, lignin, and crude protein (Hancock et al., 2014).

The USDA Agricultural Research Service handbook series on NIRS formalized the approach for forage applications and emphasized that accuracy is fundamentally calibration dependent. The analysis of a sample usually does not require extensive processing samples are typically oven-dried at 60 °C for 48-hour and ground to reduce moisture interference and particle-size effects. Sample preparation can influence measured dry matter and some composition estimates, especially for ensiled materials where drying can cause volatile losses (Haigh, 1995). This simplified process improves the efficiency and practicability of daily forage quality evaluation. In practice, NIRS calibration models are built by scanning a large, representative set of forage samples (dried and ground) for which reference analyses (wet chemistry) are available. For example, Liu and Han (2006) developed modified partial least-squares regression models from 157 wheat straw silage samples scanned over 1100–2500 nm. Using Kjeldahl crude protein and detergent fiber reference data, they achieved cross-validated correlation coefficient (R² ≈ 0.95) for CP (standard error of cross-validation (SECV) ≈ 6 g/kg DM) and R² ≈ 0.87 for DM (SECV ≈ 23 g/kg DM). Similarly, Norman et al. (2020) collected 4385 forage samples (annual/perennial grasses, legumes, forbs) and scanned them at ~1100–2498 nm. Their calibrations yielded the ration of standard error or performance: standard deviation (RPD = 5.3) for CP prediction (SECV 1.06%), RPD = 4.3 for NDF (SECV 3.5%), and RPD ≈ 3.7–3.9 for ADF and *in vitro* dry-matter digestibility. These representative studies illustrate typical NIRS calibration outcomes: high R² (calibration and cross-validation) and high RPD values for core nutritive traits under controlled conditions. Notably, these cited models were evaluated by SECV or withheld-out subsets truly independent validation sets generally yield slightly lower R² due to additional sample variation. In practice, robust forage calibrations usually have R² > 0.85 and RPD > 2–3 for key traits; RPD values above 3-4 (as in the examples above) indicate excellent predictive power, whereas RPD ~2–3 may suffice for screening purposes.

For wheat forage research and breeding, NIRS is especially valuable because it enables high-throughput phenotyping of large breeding populations, mapping panels, and multi-environment trials, where conventional wet chemistry would be prohibitively costly and slow. In contemporary forage quality evaluation, NIRS is becoming a significant tool because it can examine many samples concurrently with high accuracy (Saha and Kern-Lunbery, 2023). The nutritive analysis of forages tends to entail measurements of key chemical compounds that influence animal performance. Core laboratory traits for wheat forage quality are dry matter (DM), crude protein (CP), acid detergent fiber (ADF), neutral detergent fiber (NDF), acid detergent lignin (ADL), total digestible nutrients (TDN), relative feed value (RFV), and relative forage quality (RFQ). These traits, once calibrated, can be efficiently estimated by NIRS for routine screening and research purposes.

### 4.1 Dry matter concentration and dry matter yield

DM provides the basis for expressing forage nutrient concentrations independently of variation in moisture content and is therefore essential for comparisons among genotypes, environments, developmental stages, and management treatments. Accurate DM determination is particularly important in wheat forage because its high moisture content during vegetative growth can substantially influence estimates of available forage biomass and nutrient concentration. Oven-drying remains widely used, although losses of volatile compounds can introduce measurement bias under some conditions (Haigh, 1995). Reporting nutrient composition and feed characteristics on a DM basis equalizes the values of feedstuffs across feedstuffs by removing the variation caused by moisture, therefore allowing meaningful comparisons and allowing the development of accurate ration formulation (Ball et al., 2001).

#### 4.1.1 Significance of dry matter concentration

DM determination is not commonly considered a chemical characterization; it provides the basis for the proper evaluation of all other chemical constituents in forage. Accurate DM measurement is required to report analytical data on a total DM basis, which would make valid comparisons among various feedstuffs. Moreover, proper DM estimation is essential in the development and the balancing of ruminant diets. Winter wheat forage has a high degree of digestibility and can promote the growth of livestock. It is, however, also associated with some negative properties, such as low DM content and a tendency to cause bloat in ruminants (Fieser et al., 2007). Among small grain species cultivated for winter forage, wheat generally yields more DM yield per hectare than barley (Watson et al., 1993). Horn (2006) explains that wheat pasture bloat is associated with characteristics of lush, highly digestible forage and high rates of intake, although the risk is influenced by animal, plant, and management factors.

### 4.2 Detergent fiber fractions

As forage crops mature, fiber concentration generally increases, while digestibility, nutritive value, and intake potential decline. The detergent fiber analysis system is the most popular system of evaluation of the forage fiber in forages that is now widely used, where the content of fiber is measured. The theory of detergent fiber analysis is based on the difference in digestibility of the components of the plant cells. Plant tissues are widely classified into the less digestible cell wall fraction (fiber), which consists of cellulose, hemicellulose, and lignin, and the more digestible cell contents, which principally consist of starches and soluble sugars. The two fractions can be successfully separated with the help of special detergent solutions, namely neutral detergent (sodium lauryl sulfate and EDTA at pH 7.0) to isolate neutral detergent fiber (NDF), acid detergent (cetyltrimethylammonium bromide in 1 N H_2_SO^4^) to isolate acid detergent fiber (ADF), and stronger acid detergent (72 percent sulfuric acid) to isolate acid detergent lignin (ADL) according to the fiber analysis methodology proposed by Van Soest (2003). (**Figure 1**) shows the separation of plant cell constituents into NDF and insoluble, followed by further fractionation into ADF and ADL. The hemicellulose and cellulose are estimated via these formulas (Goering and Van Soest, 1970; Van Soest et al., 1991).

**Figure 1:**
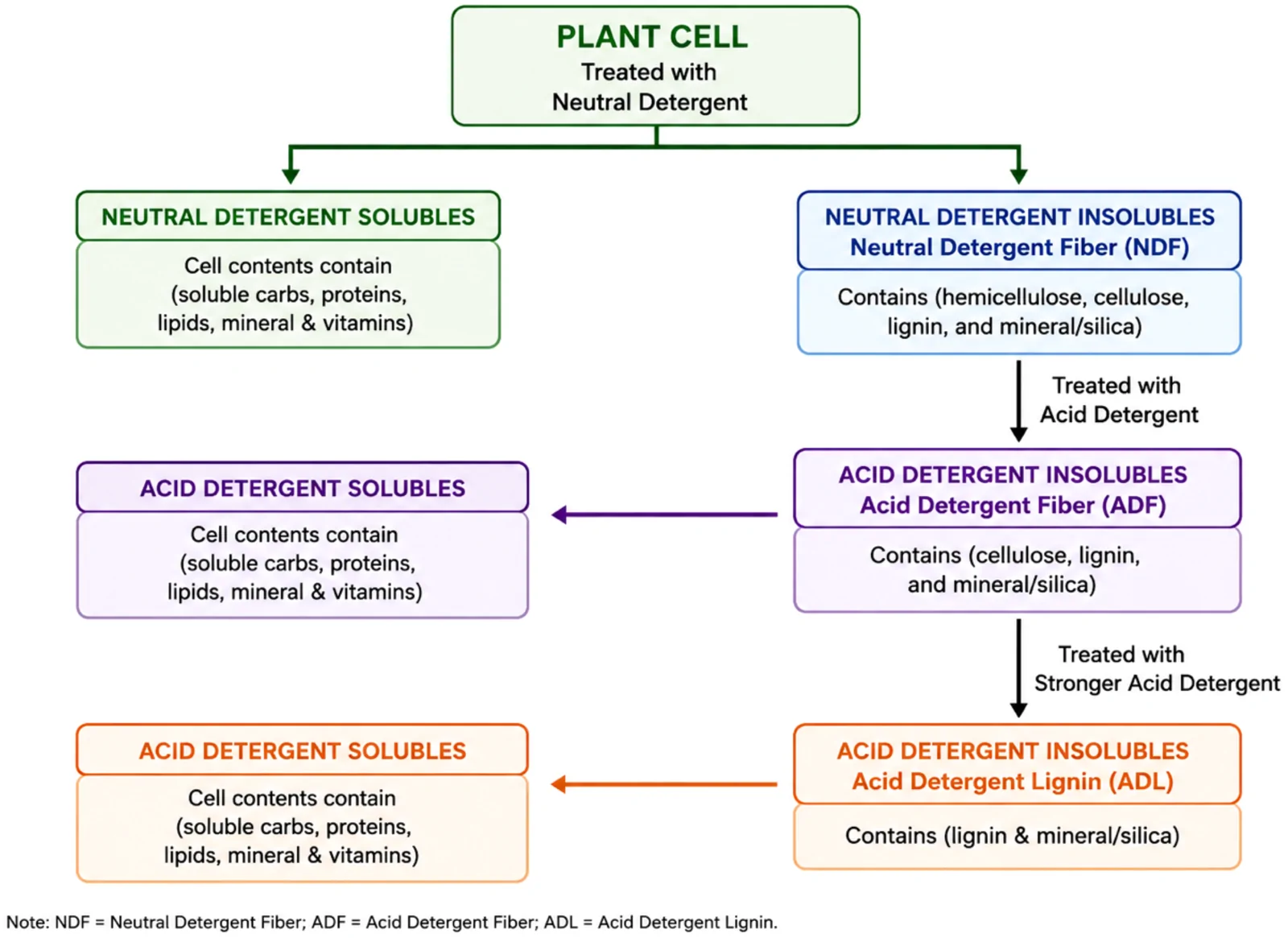
Detergent fiber fractionation of plant cell components (NDF, ADF, and ADL System, Adapted from Van Soest’s fiber analysis).


Hemicellulose(%DM)=NDF(%DM)−ADF(%DM)

Cellulose(%DM)=ADF(%DM)−ADL(%DM)

#### 4.2.1 Neutral detergent fiber and intake potential

NDF is the proportion of feed that cannot be dissolved in the neutral detergent solution, which eliminates most cell contents, including soluble carbohydrates and proteins, leaving only the fibrous structural components (Bruno-Soares et al., 1998). NDF is an efficient estimate of the total fiber content of the forage that includes the key components of the cell walls of the plants: cellulose, hemicellulose, lignin, and a small amount of cutin, silica, ash or residual protein. NDF is of paramount nutritional importance because it provides an indication of the bulkiness or physical fill of the forage (Saha et al., 2010). In the case of forage grasses, concentrations of less than 50 percent NDF are normally related to a high-quality forage along with higher intake potential, whereas concentrations above 60 percent may indicate with a poor-quality forage since they are less digestible and have lower intake potential (Van Saun, 2023).

##### 4.2.1.1 Significance of neutral detergent fiber

With an increase in the NDF content of feed and a decrease in DM intake, the chewing activity increases. In turn, forages that contain more NDF content are typically related to lower digestibility and lower consumption rates among ruminants. There is an inverse association between NDF content of forage and voluntary consumption potential of animals like cows and calves; therefore, lower NDF forage contents are usually consumed more than higher NDF ones (Biazzi et al., 2017). However, NDF concentration alone does not fully describe the feeding value of forage because the digestibility of the NDF can vary considerably among forage species and maturity stages. Therefore, NDF digestibility is commonly evaluated together with NDF concentration to provide a more accurate estimate of energy availability and animal performance. When developing a ration, this necessitates the incorporation of an adequate amount of effective NDF to promote optimum rumen activity and avoid metabolic diseases. Nonetheless, a high consumption of NDF may reduce total feed consumption and ultimately affect the performance of animals adversely. NDF can be digested variably, and it depends on the forage species as well as the maturity stage (Van Saun, 2023). Consequently, it is important to monitor the NDF levels on a regular basis to maximize the forage quality and animal performance because it can determine the time to harvest and the diet formulation to reach a desirable ratio of fiber and nutrient supply.

#### 4.2.2 Acid detergent fiber and forage digestibility

The NDF fraction may be further subjected to an acid detergent solution that will dissolve the hemicellulose fraction. ADF is the remaining residue, is the portion of plant cell walls that cannot be dissolved in acid (Fulgueira et al., 2007). ADF is mainly composed of cellulose and lignin, with other minor components of minerals and silica (Saha et al., 2010). These constituents are less digestible forage fractions. Even though cellulose can be digested slowly, the process of cellulose breakdown is significantly hindered by the existence of lignin that cannot be digested. ADF is thus only partially digestible, and its concentration is inversely correlated with overall forage digestibility; a high ADF concentration is usually associated with low digestibility. To achieve the best level of forage quality, it is suggested that ADF contents of the grasses should not exceed 35% (Van Saun, 2023). ADF is therefore usually utilized as a predictive index of forage digestibility.

##### 4.2.2.1 Significance of acid detergent fiber

Forages exhibiting lower ADF, and lignin are desired because they have better nutritive digestibility, thus increasing the consumption of forage (Biazzi et al., 2017). There are several factors that contribute to increased ADF levels in forages, such as the advanced plant maturity, great stem proportion, lignification, leaf loss, weathering, rain damage, high temperatures, and the existence of undesirable plant species or weeds (Beltzer, 2003). The ADF content thus must be monitored to determine the optimal timing of forage harvest, as fiber accumulation is strongly influenced by the stage of maturity. ADF, therefore, is a very important parameter in the research and in the process of making decisions regarding forage management, ration formulation, and general livestock productivity. Although both NDF and ADF contain lignin, the ADF fraction is generally less digestible because it excludes much of the more digestible hemicellulose component and contains a greater relative proportion of lignin and cellulose, which restrict microbial degradation in the rumen (Tamminga, 1993). This shows the significance of separating the fiber components in the assessment of the quality of forage and the formulation of feeding plans.

#### 4.2.3 Acid detergent lignin and fiber digestibility

A more acidic solution can be added to the ADF fraction to dissolve cellulose and leave lignin and silica/mineral components. This remaining fraction is termed the ADL and is widely used to measure the lignin content of individual forage samples (Van Soest, 1973). Lignin is a complex, high molecular weight organic polymer that constitutes an important structural component of plant secondary cell walls (Van Saun, 2023). Lignin is also largely concentrated in the fibrous stems, leaves, and hulls of plants, where it helps stiffen cell walls. Lignin significantly reduces cell-wall digestibility by forming chemically limiting microbial access to cellulose and hemicellulose, which limits the degradation of structural carbohydrates in the rumen (Moore and Jung, 2001; Buxton and Redfearn, 1997). The higher the level of lignin in a feed, the less the digestion of related cellulose, which leaves most of the fiber undigested by the ruminants (Beltzer, 2003). The result of this is decreased forage digestibility and nutritional value. Thus, low lignin is thought to be beneficial in enhancing the quality of forage and maximizing the nutrient content.

##### 4.2.3.1 Significance of acid detergent lignin

Lignin buildup, which is measured as ADL, is a limiting factor affecting forage quality and digestibility. This is of special concern for forages grown in the southern areas, where the conditions are characterized by the increased temperatures in the growing season leading to faster lignification of the plants. Higher levels of lignin have been linked to low digestibility of forage by ruminants, resulting in low levels of nutrients and decreased animal performance, including decreased milk production (Stokes and Prostko, 1998). Van Soest (1993) emphasized that a considerable proportion of the interspecies variability observed in the relationship between lignin concentration and DM digestibility can be attributed to interactions with NDF content. The relationship between lignin and digestibility when lignin is expressed as a proportion of the fiber fraction also seems to be more uniform across the types of forage in both legumes and grasses. Even though lignin does not add any digestible energy to livestock, it greatly reduces the digestibility of the other fiber components, especially cellulose and hemicellulose. Thus, the expression of the lignin in the form of fiber would improve the interspecies comparability of its impacts on digestibility. Moreover, legumes generally have ADL concentrations that are at least twice as high as the ones found in the grasses at similar digestibility levels. Buxton and Russell (1988) made the conclusion that the adverse influence of lignin on the digestibility of NDF is 62 percent more in grasses than in legumes. This supports the critical position of lignin in the quality of forage and the significance of ADL as the main indicator of forage assessment and breeding schemes to enhance digestibility. From a breeding perspective, ADL is a valuable secondary selection trait because lignin concentration is a major determinant of cell wall digestibility, exhibits exploitable genetic variation in wheat, and has been targeted in GWAS and GS studies; therefore, selecting genotypes with lower or optimized ADL provides a practical route to improve forage digestibility and overall feeding value (Joshi et al., 2019; Maulana et al., 2019a; Esposito et al., 2022).

### 4.3 Crude protein and crude protein value

CP is an imperative predictor of the forage nutritional value, especially when it comes to livestock feeding and ration formulation. CP, as the name suggests, is an estimate of nitrogen compounds in forage, including both protein (a combination of amino acids) and non-protein nitrogen (NPN) compounds (ammonia, nitrates, individual amino acids, and urea) (Fulgueira et al., 2007). In routine forage analysis, the amount of protein cannot be measured directly, and it is estimated using the amount of nitrogen (N) in the feed sample. Nitrogen is an essential element of protein, and even though the NPN compounds are not proteins, per se, they can be used by the rumen microbes to produce microbial protein. It is usually believed that true protein has a value of 16% N. Thus, during laboratory tests, the N level of a forage is multiplied by a standard conversion factor of 6.25 to determine CP (Van Soest, 1994).


CP(%DM)=N(%DM)×6.25

Nevertheless, the assumption is not necessarily true because the actual content of forage proteins in terms of N can vary (Cherney, 2000). Despite this inconsistency, CP remains a useful parameter for preliminary forage quality assessment. Accurate evaluation of metabolizable protein, defined as the fraction of dietary protein absorbed in the small intestine, is essential for precise ration formulation, particularly when high-quality forages contribute substantially to the dietary protein supply (Weiss and Hall, 2020). Although there is a notable difference in CP concentrations between feed types, the higher the CP content within a particular forage are typically associated with improved nutritional quality. For instance, CP content may vary widely across a population of forage species, often ranging from 12-26% of DM depending on the wheat growth stage, N and cultivar, tends to have a higher CP in comparison with other cool-season grasses (8-23%) (Kim and Anderson, 2015; Castillo and Davidson, 2024).

#### 4.3.1 Significance of crude protein

CP is one of the basic parameters to determine the protein level of forage crops, and it is crucial to determine the quality of forage crops. However, excessive focus on CP may contribute to the inaccuracy of overall forage quality, especially when the energy needs are not met. They should pay more attention to the digestible energy content of the forage as opposed to the CP levels, and in most cases, the latter is the most constraining nutritional component of the livestock diet. Although it is important to analyze the total or CP content of the feedstuff (e.g., forages, silages, or grains), protein may not be adequate to meet the nutritional requirements of different livestock classes. Protein supplementation may be required when forage CP or metabolizable protein supply is insufficient for the targeted class of livestock and level of production. The use of CP is thus deceptive as an indicator of forage quality. Nutritional adequacy requires a balance of nutrients, and an excess of one nutrient cannot be used to replace deficiencies in other nutrients. CP values should be interpreted with respect to a variety of agronomic and physiological conditions, such as the species of the plant and its maturity, fertilization, and the content of the fibers (especially NDF and ADF). When the forages grow and mature, their concentrations of CP normally decrease because of dilution by rising structural fiber content (Fulgueira et al., 2007; Weiss and Hall, 2020). Besides, this process may be changed by fertilization, which is why it is necessary to interpret CP in relation to other quality indicators, especially fiber fractions. Protein needs of most livestock classes are quite low in the southeastern (SE) region of the U.S. Protein requirements vary by livestock class, body weight, physiological stage, and production goal. For example, beef cows require greater protein supply during early lactation than during maintenance or non-lactating periods because milk production increases nutrient demand (NASEM, 2016; Troxel et al., 2018). As an example, the demand of CP of beef cows is the highest at about 12% in early lactation and decreases to about 7% in non-lactating cows (Hancock et al., 2014). This also makes the point that matching forage quality measurements to the nutritional requirements of the targeted livestock group should be done and not just CP as an independent measure. In wheat forage, CP concentration is generally highest during vegetative growth and declines with advancing maturity, particularly as plants progress through stem elongation, heading, and grain filling, because leaf proportion decreases while stem development, structural tissue, and fiber/lignification increase (Edmisten et al., 1998; Ronga et al., 2020; Kubesch, 2024).

### 4.4 Calculated energy values

Calculated energy values of forages are assumed to be the metabolizable energy (ME)that livestock can obtain from a certain forage source. It has been well known that the energy content of forage is negatively correlated with the level of fiber content, especially NDF and ADF. The direct determination of the feed energy by animal metabolism trial needs sophisticated equipment and is time-consuming and expensive. This has resulted in coming up with predictive equations that can be used to predict the energy content based on easily measurable forage parameters, which include CP, fiber fractions (ADF, NDF), and DM content. Total digestible nutrients (TDN), relative feed value (RFV), and relative forage quality (RFQ) are among the indicators of the forage energy value that can be obtained as a result of regular core forage analysis (Stokes and Prostko, 1998).

#### 4.4.1 Total digestible nutrients and forage energy value

TDN is the digestible fraction of the feed and is usually referred to estimate of digestible energy value of forages, calculated from the digestible fractions of protein, fiber, nonfiber carbohydrates, and fat. TDN is usually expressed as a percentage and can be calculated from ADF content using the equation below (Undersander et al., 1993; Beltzer, 2003; Zhang and Rocateli, 2017):


TDN(%)=88.9−(0.79×ADF)

Alternatively, in situations where the compositional data particularly digestible components are more detailed, TDN may be calculated as the total effect of the digestible components of the feed, which includes digestible CP, digestible fiber, digestible non-structural carbohydrates (starches and sugars), and digestible fats with a multiplier of 2.25 to reflect the fact that the energy content of fats is 2.25 times higher than that of other constituents in the feed (Fulgueira et al., 2007). Direct comparison is possible in terms of TDN values of forage of different crops belonging to the same crop species. The comparison of species, however, is more complicated compared to comparisons within species because of inherent differences in nutrient composition and digestibility. The forage maturity also affects TDN content, with more mature forages generally (greater fiber content) having lower TDN values. In addition, the content of TDN differs between forage types. Reported TDN values vary considerably among forage species; however, direct comparisons should be interpreted with caution because forage nutritive value is influenced by multiple interacting factors, including species, genotype, developmental stage at harvest, environmental conditions, and harvest and storage practices. Furthermore, TDN estimates are inherently dependent on the prediction equations or analytical methodologies used for their calculation, which can contribute to variation among studies (Ball et al., 2001; Weiss et al., 1992; Coblentz and Walgenbach, 2010). An example is that the alfalfa usually has a higher value of TDN (60-70%) than that of wheat (57-69%) and other cool-season grasses (55-68%).

##### 4.4.1.1 Significance of total digestible nutrients

TDN is an older method of estimating the energy value of forage and feedstuffs and was in common use before the more sophisticated systems, including Net Energy, were adopted. The idea was initiated during the initial test of digestibility in the Weende Experimental Station of the University of Gottingen in Germany, conducted before 1860 (Schneider and Flatt, 1975). These pioneering works formed the framework on which the TDN system was developed, which later turned out to be a common tool for assessing the feed energy content and constructing the livestock rations. Until it was phased out in favor of the Net Energy for Lactation system developed by the National Research Council in 1971 (NRC, 1971), TDN was widely used, especially in the U.S., to determine the nutritional requirement of dairy cows. Although this change has occurred, TDN has continued to be popular in most areas because it is convenient in the evaluation of feeds and formulation of ratios (Ball et al., 2001). Even though TDN remains a useful measure, it has some limitations. The main weakness is that it does not account for some energy losses, especially heat increment and, to a lesser extent, gaseous losses, which are very high in ruminant systems. Consequently, TDN is prone to inflating the energy value of fibrous forages compared to high starch contents like grains (Van Soest, 1994; Weiss and Hall, 2020). The high TDN values point to the increased energy availability, which is positively correlated to increased animal performance, such as weight gain, milk yield, and reproductive efficiency (Moore and Undersander, 2002). Furthermore, TDN is commonly used in the quality of forages and in economic evaluation of feed resources. TDN is a more detailed parameter of the nutritive value of various forages by incorporating digestibility and nutrient composition and assists in informed decision-making in animal production systems (Ball et al., 2001).

#### 4.4.2 Relative feed value

Ranking forage lots can be difficult because forage quality depends on multiple interacting traits, including digestibility, intake potential, fiber concentration, protein concentration, and energy availability. RFV is an index that is dimensionless index calculated from ADF and NDF, which ADF can be interpreted as the digestibility and NDF as intake potential. The more forage maturity progresses, the higher the fiber content, which leads to a lower RFV (Hackmann et al., 2008). RFV is commonly used to compare forage lots based on estimated digestibility and potential DM intake, particularly for hay quality assessment and market grading (Stokes and Prostko, 1998). Even though RFV does not include units, it offers a useful and standardized value for comparing the nutritive value of various lots of forage. To illustrate, forages with an RFV of more than 100 will be deemed as being of good quality as compared to the mature (full bloom) alfalfa hay, whereas those that fall below 100 will be considered as having low quality (Ball et al., 2001). This index is useful in the pricing of forage, marketing, and the formulation of rations because it is simple and comparable. RFV is used to combine several quality considerations, such as fiber content, intake potential, and digestibility, into one numerical value. The RFV index is standardized relative to full-bloom alfalfa hay, which is assigned a reference value of 100. Forage lots with RFV values above 100 are predicted to provide greater digestible DM intake potential, whereas values below 100 indicate comparatively lower forage quality. This is calculated as per the methodology of Saha et al. (2010) and Zhang and Rocateli (), as shown below:

RFV = (DDM × DMI)/1.29 (Saha et al., 2010) or RFV = DDM × DMI × 0.775 (Zhang and Rocateli, ())

Where,

- DDM (% digestible DM) = 88.9 - (0.78 × ADF%)
- DMI (% DM intake as % of body weight) = 120/NDF%

##### 4.4.2.1 Significance of relative feed value

RFV is also employed in the forage market to draw a comparison involving the quality of hays and other forages, which gives an approximation of their capabilities to satisfy the nutritional needs of livestock (Putnam, 2004; Fulgueira et al., 2007). The increased RFV indicates better forage quality, as it suggests increased digestibility and intake potential (Ball et al., 2001). Forages fall into quality grades based on RFV scores, whereby the highest quality is Pride of Place quality (Prime), and the lowest quality is Grade 5 (Stokes and Prostko, 1998). This classification allows standard pricing and quality evaluation in the commercial forage sales. Even though RFV is useful, it has significant limitations. The index is calculated on the assumption that NDF (DMI) and ADF (DDM) are fixed relative to each other. Nevertheless, forages with a similar NDF concentration can differ in digestibility owing to changes in fiber and plant structure. This means that RFV can fail to appreciate the quality of highly digestible forage by underestimating its actual digestive capacity (Saha et al., 2010). Also, a large RFV is not always associated with high milk production or animal performance because other nutritional aspects like ME, protein degradability, and mineral balance also determine the productivity. Caution should be exercised when comparing RFV values of various sources since there are various equations and calculation techniques available, and this may result in the inconsistency of results (Stokes and Prostko, 1998).

#### 4.4.3 Relative forage quality

The RFQ was proposed by scholars at the University of Wisconsin as an alternative to RFV to improve forage ranking by incorporating estimates of total digestible nutrients and fiber digestibility (Undersander et al., 2002). It is determined by two main parameters, DMI and TDN, calculated using formulas that are more realistic in reflecting livestock forage utilization. In contrast to RFV, which uses DDM estimated from ADF, RFQ uses estimated TDN, which can incorporate digestible fiber and other nutrient fractions. Although RFQ has a similar purpose as RFV, ranking forages by their relative nutritive value, it has some improvements. Like RFV, RFQ is a dimensionless index and are expressed as unitless values whose base index is 100, which represents full-bloom alfalfa, and are used to be able to compare two or more forages of a similar type. Similar to RFV, RFQ allows considering potential energy intake and is useful in the context of forage and marketing.

The RFQ is calculated using the formula:


RFQ=[DMI(% of body weight)]×[TDN(% of DM)]÷1.23

This formula is the same as that of RFV except that TDN is used in place of DDM, an adjustment factor of 1.23 is used rather than 1.29, and the reference value of 100 is still used for full-bloom alfalfa. Although RFQ was designed to produce values similar to RFV, the two indices are not directly interchangeable. Unlike RFV, which is based on DDM, RFQ incorporates TDN, accounts for NDF digestibility, and uses species-specific equations for legumes and grasses (Undersander et al., 2002; Moore and Undersander, 2002).

##### 4.4.3.1 Significance of relative forage quality

RFQ represents an improved forage assessment index that aims at addressing the shortcomings of the RFV system. By integrating the NDF digestibility into estimates of both DMI and total TDN energy, RFQ provides a more reliable reflection of the overall forage nutritive value and its potential contribution to animal performance (Undersander et al., 2002). This approach improves the accuracy of forage evaluation, particularly for highly digestible forages where RFV may underestimate feeding value. The other main advantage of RFQ is its ability to better distinguish forage types with different fiber digestibility characteristics, which the RFV model does not take into account. Because fiber digestibility varies substantially among forage species, especially between grasses and legumes, RFQ provides a more accurate ranking of forages based on their feeding value. This improved discrimination enhances forage pricing, marketing, and ration formulation decisions in livestock production systems (Undersander et al., 2002). Despite these advantages, RFQ has some limitations. For example, the index is not recommended for evaluating corn silage because it does not account for variation in starch digestibility, which is a major contributor to the energy value of silage-based diets. Nevertheless, RFQ has been shown to correlate more strongly with animal performance indicators, such as milk production, than RFV, making it a more comprehensive tool for assessing forage quality in both research and practical feeding systems (Moore and Undersander, 2002). Because wheat is a cool-season cereal grass, RFQ estimates for wheat forage should be based on equations appropriate for cool-season grasses rather than legume-specific equations.

## 5 Nutritional and management significance of wheat forage quality

Winter wheat forage (pasture) is highly valued in cool-season grazing systems because young, vegetative wheat pasture can provide high CP, digestible energy, mineral content, palatability, and digestibility relative to many other forages, especially in cool-season grazing systems. Wheat forage can provide substantial digestible energy, particularly when grazed during vegetative growth, because young wheat pasture is typically high in CP commonly range between 20-30% of DM (sometimes exceed 30% depending on growth stage), low in fiber, and described as a high-energy forage for grazing livestock (Lollato et al., 2017; Ball et al., 2001; Duncan et al., n.d; Beck and Jennings, n.d.; Kim and Anderson, 2015). Extension guides describe wheat forage as “high in protein, energy, and minerals, and low in fiber” when grazed vegetatively, it can exceed 80% *in vitro* digestibility and provides palatable and succulent feed (Duncan et al., n.d). In practical terms, this means stocker cattle gains on wheat pasture often reach 0.7-1.3 kg/d under good conditions, even un-supplemented steers have averaged 0.5-1.8 kg/d weight gain on wheat (Gunter and Combs, 2019; Fieser et al., 2007; Duncan et al., n.d). Overall, forage quality is very high early in the season: for instance, harvested wheat forage often meets or exceeds quality benchmarks of<50% NDF and<30% ADF that are recommended for maximal intake and digestibility (Atis et al., 2026).

Besides protein and energy, wheat forage is a rich source of minerals, although the mineral balance may require management. Research has shown that wheat pasture generally supplies adequate phosphorus (P) and magnesium (Mg) but often contains very high potassium (K) and very low calcium (Ca), particularly during rapid spring growth (e.g. >4% K and ~0.2% Ca on a DM basis) (Gunter and Combs, 2019; Fieser et al., 2007). Calcium is therefore typically the limiting mineral for grazing livestock on wheat pasture (Gunter and Combs, 2019). Producers should therefore provide a calcium-rich mineral supplement when animals graze actively growing wheat. Because wheat plants typically contain 80 to 90% moisture, DM intake may be limited, so livestock may benefit from access to dry hay or grain to meet their energy requirements (Duncan et al., n.d).

Another important aspect of wheat forage is its seasonal adaptability. Wheat grows vigorously during the cool season, particularly in fall and early spring, when warm-season grasses and many perennial forages are dormant or have limited growth (Lollato et al., 2017; Duncan et al., n.d). Consequently, wheat pasture can fill an important seasonal forage gap and reduce dependence on stored feeds. In dual-purpose systems, this advantage is particularly important because the crop can provide substantial forage biomass for livestock while retaining the potential for subsequent grain production when grazing intensity and termination are appropriately managed (Gunter and Combs, 2019).

Despite its high nutritive value, wheat forage can present several nutritional and animal-health challenges. Drought stress and excessive N fertilization can promote nitrate accumulation, potentially reducing animal performance and, at sufficiently high concentrations, causing toxicity (MacKown and Northup, 2010). Nitrate toxicity in grazing cattle occurs primarily after nitrate in forage is converted by rumen microorganisms to nitrite. Under normal conditions, nitrite is further reduced to ammonia and incorporated into microbial protein; however, when nitrate intake is excessive or conversion to ammonia is overwhelmed, nitrite accumulates, is absorbed into the bloodstream, and oxidizes hemoglobin to methemoglobin, which cannot effectively transport oxygen (Thompson, 2026; Kallenbach and Evans, 2026). The resulting methemoglobinemia can cause tissue hypoxia and clinical signs such as reduced feed intake, poor growth or weight gain, weakness, ataxia, muscle tremors, rapid or difficult breathing, cyanotic or brownish mucous membranes, reproductive losses, abortion, collapse, and death in severe cases (Thompson, 2026; Kallenbach and Evans, 2026). Therefore, nitrate risk in wheat forage should be evaluated by laboratory testing, especially after drought, cloudy or cold weather, frost injury, rapid regrowth, or high N fertilization. Results should clearly specify whether nitrate is reported as nitrate or nitrate-N, because nitrate values are approximately 4.43 times nitrate-N values; for example, 2,500 ppm nitrate is equivalent to about 550 ppm nitrate-N on a DM basis (Kallenbach and Evans, 2026). For consistency, forage nitrate concentrations should be reported as ppm or mg kg^−1^ on a DM basis, while nitrite should be reported separately as nitrite or nitrite-N if measured, because nitrite is the more toxic intermediate and should not be confused with nitrate (Thompson, 2026; Kallenbach and Evans, 2026). Additionally, the mineral composition of rapidly growing wheat can also create nutritional imbalances, while lush immature pasture may increase the risk of frothy bloat (Gunter and Combs, 2019; Fieser et al., 2007). Fresh wheat forage contains substantial concentrations of soluble proteins and fermentable carbohydrates, and conditions that increase the availability of rapidly fermentable substrates may further increase bloat risk (Beck, 2023). These limitations demonstrate that the nutritional value measured in the forage itself does not necessarily translate directly into animal performance without appropriate grazing and nutritional management.

From a management perspective, wheat forage quality should be interpreted as the outcome of bothplant composition and grazing conditions rather than as a fixed property of the crop. Youngvegetative wheat can provide high crude protein, digestible energy, palatable forage, and relativelylow fiber, but animal performance depends on how the forage is managed, including forage availability, stocking rate, fertility, cultivar, and environmental conditions (Duncan et al., n.d; Beck and Jennings, n.d.; Lollato et al., 2017). High moisture concentration in lush wheat pasture can make it difficult for grazing livestock to meet daily dry matter intake needs, so dry forage or grain supplementation may improve performance under some conditions (Duncan et al., n.d; Beck and Jennings, n.d.). Rapid growth, excessive nitrogen fertilization, drought stress, high soluble nutrients, and mineral imbalance can also increase the risk of nitrate accumulation, frothy bloat, or mineral disorders under certain conditions (MacKown and Northup, 2010; Gunter and Combs, 2019; Bartley et al., 1975; Gadberry et al., 2022). Therefore, effective use of wheat pasture requires coordinated management of grazing stage, forage allowance, stocking rate, soil-test-based fertilization, nitrate testing when risk is high, and appropriate mineral or bloat-prevention supplementation (Beck and Jennings, n.d.; Lollato et al., 2017; Bartley et al., 1975; Gadberry et al., 2022). This integrated approach allows producers to capture the high feeding value of wheat forage while reducing nutritional and animal-health risks.

## 6 Agronomic, environmental, and genetic factors affecting wheat forage quality

Wheat forage is an important source of high-quality forage for livestock production systems. Forage quality is generally defined as the combination of characteristics that influence animal performance, including nutritional value, voluntary intake, palatability, and digestibility. Numerous factors influence forage quality; however, the most critical determinants are plant maturity at grazing or harvest, genotype/cultivar, and the growing environment. Additional factors include genetic variation among wheat varieties, soil fertility, environmental conditions such as temperature and moisture during forage growth, fertilization practices, and the incidence of pests and diseases (Amigot et al., 2006; Hilario et al., 2017). These factors influence wheat forage quality mainly by altering leaf-to-stem ratio, biomass accumulation, CP concentration, fiber deposition, lignification, mineral composition, and digestibility.

### 6.1 Growth stage at grazing or harvest

Plant growth stage at grazing or harvesting is one of the most influential factors affecting wheat forage quality because it strongly determines nutritive value, digestibility, intake potential, and biomass yield (Vaughn et al., 2024); Therefore, identifying the appropriate grazing or harvest stage is essential for balancing forage quantity, forage quality, animal performance, and profitability (Sadeghpour et al., 2022). Phenological stage models of growth have been developed to describe the developmental stages of wheat (Zadoks et al., 1974). The Zadok’s scale is an elaborate numerical development staging system that is widely used by plant breeders and agronomists to describe the phenological development of cereal crops. It provides a more specific and standardized system than more general developmental systems like booting, flowering, milk and soft dough. These frameworks are used to associate changes in forage quality parameters with numerical data on plant maturity, enabling a systematic assessment of quality dynamics throughout the growth cycle. In practical terms, the maturity stage determines the balance between (1) forage quantity (biomass accumulation) and (2) forage quality (CP concentration, ADF, NDF, digestibility, DM, mineral supply, and fermentable carbohydrate availability). Numerous studies have shown that as wheat forage advances to later maturity stages, CP concentration declines, whereas NDF, ADF, and ADL concentrations increase (Sadeghpour et al., 2022; Kim and Anderson, 2015; Capstaff and Miller, 2018). This trade-off is central to profitability in wheat-based forage systems. The optimal harvest or grazing window depends on production goals, including the target is high daily gain on pasture, conserved forage quality for lactating animals, maximum tonnage for winter feed inventories, or dual-purpose integration with grain production (Shroyer et al., 1990). Evidence from wheat pasture systems consistently shows that vegetative forage is exceptionally nutrient-dense, however its quality declines rapidly as plants advance toward heading and grain development. For instance, extension guidelines from Kansas State University report that *in vitro* DM digestibility often decreases from ≥80% in vegetative stages to<60% by the soft-dough stage, while CP declines sharply across the same developmental period (Shroyer et al., 1990). Another study by Lyon et al. (2001) reported that winter wheat forage grown in Nebraska had mean CP concentrations of 310 g kg^−1^ DM, equivalent to 49.6 g N kg^−1^, at fall harvest and spring jointing. However, CP declined to 170 g kg^−1^ DM, equivalent to 29.8 g N kg^−1^, when forage was harvested at the boot stage (Zadoks 41). (**Table 1**) illustrates a pronounced decline in CP in wheat forage with advancing maturity, while an increase in fiber components, including ADF, NDF, and lignin. The ranges in **Table 1** are drawn from several studies of forage wheat (Collar and Aksland, 2001; Lyon et al., 2001; Filya, 2003; Bailey et al., 2007; Xie et al., 2012; Mullenix et al., 2014; Kim and Anderson, 2015). Several biological mechanisms explain why wheat forage quality changes predictably with maturity.

**Table 1:** Nutrient composition of wheat forage as affected by maturity (Zadoks’s growth scale).

Maturity level of wheat		CP		ADF		NDF		Lignin	
Zadoks growth scale –––––––––––––––––%––––––––––––––––				
28 - Main stem and eight tillers 30 - Pseudostem 32 - Second node detectable 37 - Flag leaf just visible	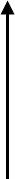	∼19-27 ∼19-25 ∼13-23 ∼12-20	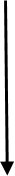	∼15-21 ∼20-28 ∼21-32 ∼24-34	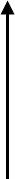	∼39-45 ∼40-48 ∼43-54 ∼44-55	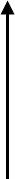	∼4 ∼4-5 ∼4-6 ∼4-7	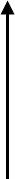

#### 6.1.1 Morphological shift and leaf: stem ratio

As wheat advances developmentally, the relative proportion of leaf tissue typically declines compared with stems and eventually, reproductive structures. Because leaves generally contain more cellular contents and lower levels of structural cell wall components per unit DM than stems, a reduced leaf: stem ratio is typically associated with reductions in CP and digestibility, along with increases in fiber-related limitations (Ball et al., 2001; Collins et al., 2017).

#### 6.1.2 Cell-wall deposition and lignification

As wheat plants mature, wheat tissues accumulate more structural carbohydrates deposition and lignin in the cell wall matrix. Consequently, even when total NDF does not increase consistently, the digestible fraction of NDF often declines because increased lignification expands the indigestible component of the cell wall (Ball et al., 2001).

#### 6.1.3 Nitrogen dilution and carbohydrate partitioning

N concentration in wheat tends to decrease during the growing season mainly because the rate of carbon fixation and biomass accumulation exceeds that of N assimilation (Ball et al., 2001). As a result, CP typically decreases during plant development due to the dilution effect caused by increasing biomass and carbohydrate deposition in vegetative tissues, along with the progressive partitioning of nutrients toward reproductive spikes (Virgona et al., 2006; Collar and Aksland, 2001). In cereals, the grain-filling stage promotes substantial starch accumulation, which increases nonstructural carbohydrate reserves and may dilute certain fibers at the whole-plant level. This dilution phenomenon has been widely reported for small-grain forages and contributes to the distinct maturity-related changes in forage composition observed in cereals compared with legumes such as alfalfa (Collar and Aksland, 2001; Ball et al., 2001).

### 6.2 Forage species and botanical composition

Forage quality is strongly shaped by botanical composition because plant species differ in their dominant structural tissues, cell-wall composition, N dynamics, seasonal growth patterns, and responses to defoliation. These species-specific differences become particularly relevant in mixed crop-livestock production systems, where wheat is often evaluated alongside alternative winter forages, multi-species forage mixtures developed to extend grazing periods, and rotations that integrate cool-season annual forages (wheat) with warm-season perennial pastures that differ fundamentally in quality and digestibility constraints (Collins et al., 2017). There is a significant variation in forage quality among legumes and grasses, cool-season (C3) and warm-season (C4) grasses, and wheat relative to other cool-season annual grasses.

#### 6.2.1 Legumes versus grasses

Across temperate forage systems, legumes generally contain higher CP than grasses, although CP in both declines with advancing maturity as cell-wall (fiber) fractions accumulate (Collins et al., 2017). Typically, CP ranges from approximately 15-25% for legumes and 10-20% for grasses on a DM basis, with concentrations decreasing as plants advance in maturity (Castillo and Davidson, 2024; Collins et al., 2017). In addition to their nutritive advantages, legumes also provide a systems-level benefit through symbiotic biological N fixation. Despite the nutritional advantages of legumes, grasses often provide agronomic stability that is particularly relevant when wheat is compared with alternative forage options. Grasses tend to offer greater persistence under frequent defoliation, improved ground cover, and more stable forage production under variable environmental conditions. These characteristics help maintain forage availability even when the nutritive profile differs from that of legumes. Consequently, many livestock producers adopt legume-grass mixtures to integrate complementary strengths by enhancing CP yield and improving composite forage quality (Xu et al., 2023). Wheat forage is often evaluated within a decision framework where legumes serve as a protein benchmark, whereas grasses provide the baseline forage. Therefore, evaluating wheat forage quality should not only consider CP and fiber fractions but also its role in filling seasonal feed gaps and its compatibility with legume integration to address protein and N constraints within the forage production system (Castillo and Davidson, 2024).

#### 6.2.2 Cool-season versus warm-season grasses

Wheat is classified as C3 grass, and many of the nutritional advantages found in wheat pastures relative to warm-season pastures reflect physiological and compositional differences between C3 and C4 forage species (Ball et al., 2001). Lee et al. (2017) reported that forage nutritive value generally declined under higher temperatures and improved with nitrogen fertilization, indicating that both climate and fertility management can modify grass forage quality. In many forage systems, cool-season C3 grasses are often associated with higher nutritive value than warm-season C4 grasses, although this relationship varies with species, maturity stage, management, and environment. Wheat forage is generally utilized during winters or early spring when warm-season forages are dormant, so this type of temperature-quality linkage is highly relevant to wheat. Notably, warm-season C4 grasses often have anatomical and cell-wall characteristics, including a greater percentage of lignified tissues (often exhibit anatomical characteristics) that reduce the ability of fibers to degrade (Mertz and Brutnell, 2014). However, a study by Ritz et al. (2020) compared cool- and warm-season grasses and reported similar NDF and ADF values.

#### 6.2.3 Wheat relative to other cool-season annual forage grasses

Within cool-season annual cereal forages, wheat is commonly evaluated against cereal rye, oat, and triticale, with small-grain forages tending to have similar nutritive values when harvested or grazed at comparable maturities, emphasizing that management aiming to maintain vegetative growth supports high quality across species (Lemus, 2021). According to Nebraska extension forage specialist Anderson (2008), wheat may be preferred dual-purpose system because it provides high-quality forage when harvested or grazed prior to stem elongation and can have potential to outperform other small-grain species such as rye and triticale. Comparative studies among small-grain forages have also highlighted structural composition and feeding value differences in species. For instance, the New Mexico State University Extension guide indicates that rye generally contains higher fiber and lower digestibility and palatability than wheat, triticale, barley, and oat hay or silage due to a greater proportion of stem tissue (Marsalis, 2018). These practical observations are consistent with broader forage management recommendations suggesting that small-grain species mixtures can be used to extend the grazing window and moderate seasonal fluctuations in forage supply (Mullenix et al., 2014). However, mixing different species may introduce management trade-offs, such as herbicide selectivity and differential maturity among species (Lemus, 2021). According to West et al. (2024), the nutritive value of wheat forage, including NDF, ADF, and CP, are generally comparable to those reported for annual ryegrass and other small-grain forages commonly grazed in the SE U.S. Similarly, Mullenix et al. (2014) reported NDF values ranged from 42 to 47% and an average CP of about 17% in annual ryegrass, triticale, and wheat pastures, which also shows comparable nutritional values among these cool-season forage species.

Direct comparison of wheat with other cool-season annual forage grasses should nevertheless be interpreted cautiously because feeding value depends not only on species but also on developmental stage and the form in which the crop is utilized. Wheat pasture is typically most valuable during vegetative growth, when CP and digestibility are high and fiber concentration is relatively low. In contrast, barley, oat, rye, and triticale may be utilized as pasture, hay, whole-crop silage, or straw, and their relative feeding value changes substantially with advancing maturity as fiber and lignin concentrations increase and grain starch accumulates. Consequently, comparisons among cereal forages are most meaningful when made within comparable developmental stages and use categories, such as vegetative pasture with vegetative pasture, whole-crop silage with whole-crop silage, and straw with straw, rather than across unrelated harvest stages or conservation methods (Coblentz and Walgenbach, 2010; Marsalis, 2018; Mullenix et al., 2014). We compiled forage yield and quality metrics for wheat, barley, oats, rye, and triticale from published sources (Edmisten et al., 1998; Collar and Aksland, 2001; Filya, 2003; Arzadun et al., 2006; Bailey et al., 2007; Anderson, 2008; Mullenix et al., 2014; Anderson, 2015; Kim and Anderson, 2015; Collins et al., 2017; Capstaff and Miller, 2018; Joshi et al., 2019; Ritz et al., 2020; Lemus, 2021; Castillo and Davidson, 2024; Kubesch, 2024; Castillo et al., 2025). Values are on a DM basis unless noted (**Table 2**).

**Table 2:** Quantitative comparison of forage yield, nutritive value, and animal performance of wheat and other cool-season annual forage grasses.

Crop	Form/growth stage	Yield (t DM/ha)	CP (% DM)	NDF (% DM)	ADF (% DM)	ADL (% DM)	WSC (% DM)	Energy (ME MJ/kg DM)
Wheat	Pasture/vegetative	~2-4	~10-12	~50-60	~30-35	~4-5	~10	~9-10
	Whole plant Silage	~5-8	~9-12	~50-60	~30-35	~4-5	~5-10	~9-10
	Hay/boot stage	~4-6	~5.4	~54.2	~39.2	~5.6	–	–
	Straw/post-harvest	~3-5	~4.2	~77.5	~50.0	~7.2	~1	~7-8
Barley	Pasture/vegetative	~3-6	~11	–	–	–	–	–
	Whole plant Silage	~4-7	~10.2	~47.6	~29.6	~4.4	~3.5	~8.7
	Hay/milky stage	~4-6	~7-10	~50-60	~35-40	~2.3	–	–
	Straw/post-harvest	~2-4	~3.8	~80.5	~48.3	~6.5	–	~8-9
Oats	Pasture/vegetative	~7-10	~12-13	~54-56	~36-37	~4,5	~7.1	–
	Whole plant Silage	~7-10	~9.5	~53.4	~35.9	~4.1	–	–
	Hay/boot stage	~4-6	~8-12	~50-60	~35-40	~4.2	–	–
	Straw/post-harvest	~2-3	~3-5	~75-85	~45-55	~6.6	–	–
Rye	Pasture/vegetative	~3-5	~12-15	–	–	–	–	~9-10
	Whole plant Silage	~4-6	–	–	–	–	–	–
	Hay	~4-6	–	–	–	–	–	–
	Straw/post-harvest	~2-3	~3-5	~70-80	~40-50	~9.0	–	–
Triticale	Pasture/vegetative	~3-6	~12.4	~49.8	~30.5	~3.8	–	–
	Whole plant Silage	~4-6	–	–	–	–	–	–
	Hay	~4-6	–	–	–	–	–	–
	Straw/post-harvest	~2-4	~3.2	~75.1	~47.5	–	–	~7.2

CP, crude protein; NDF, neutral detergent fiber; ADF, acid detergent fiber; ADL, acid detergent lignin; WSC, water-soluble carbohydrates; ME, metabolizable energy.

### 6.3 Wheat varieties

There is variability in forage quality across wheat varieties because it influences phenology, canopy structure, tissue composition, and the timing of phenological transitions that affect forage quality. In a study by Kim and Anderson (2015) dual-purpose production demonstrated that cultivar choice affects not only forage yield but also the quality profile available during the grazing window. Direct comparisons among winter wheat varieties demonstrate that forage yield and nutritive value differ among cultivars, and that these differences interact with cutting/grazing timing and environment. In a three-year field study by Kim and Anderson (2015) reported significant effects of varieties on CP were reported, with mean CP ranging from 20.8 to 24.2% across 15 winter wheat varieties evaluated. West et al. (2024) reported modest variation in CP (1.6% units P ≥ 0.03) and ADF values in their study on evaluating the nutritive value and dual-purpose potential of four wheat varieties; however, CP levels were considered adequate to meet the nutritional requirements of lactating cows and to support additional weight gain in growing calves. A major varietal difference in CP, ADF, and NDF were also revealed by Kim and Anderson (2015), where they showed that the genotype of wheat has an impact on forage yield and quality. Wheat varieties differ in vernalization requirement and photoperiod sensitivity, which makes differences in developmental progression that determine when forage remains leaf-dominant and highly digestible compared to the case when stems and structural carbohydrates accumulate. In wheat dual purpose management system, first hollow stem (FHS) is widely considered a critical stage for grazing or clipping termination to get a high-quality forage and protect subsequent grain yield. FHS is identified when a hollow internode of about 1.5 cm forms just below the developmental head (Lollato et al., 2017). Therefore, variability in FHS timing also functions as genotype-driven differences in spring developmental rate and grazing-window length (Lollato et al., 2025). These tissues occur in varying proportions across plant organs, including leaves, stems, roots, flowers, and fruits, and each contributes differently to forage quality. Ruminant digestion rumen microbes have a wide spectrum of degradability of these tissues; thick-walled xylem cells are very resistant to degradation, and their degradability is close to 0%, but mesophylls are practically all degradable. Therefore, anatomy composition and cellular characteristics both play a very important role in determining the quality and digestibility of forage of wheat genotypes.

### 6.4 Soil fertility and fertilizer management effects on wheat forage quality

In wheat managed primarily for forage and dual-purpose production system, it is a usual agronomic practice to raise the level of fertilizer application when planting to achieve both high forage biomass and high-quality forage for livestock. Such essential nutrients as N, P, K, Ca, and sulfur (S) are typically available nutrients, although of all these nutrients, N, Ca, and P are commonly the most frequent limiting nutrients for wheat forage production (Lollato et al., 2017; Gunter and Combs, 2019). One of the key nutrients is N, which often has most direct and consistent effect on CP and post-grazing regrowth, whereas response on NDF, ADF, and digestibility was smaller and strongly dependent on sampling date and maturity stage (Bailey et al., 2007). A study by Li et al. (2015a) reported that increasing N can enhance nutritive value of wheat up to an optimal level, while also increasing safety risks. In their experiment on evaluating wheat for silage across 0 to 300 kg N ha^−1^, CP and *in vitro* DM digestibility increased up to ~225 kg N/ha, while NDF and ADF tended to decline with increasing N. However, excessive N also increases plant nitrate-N content and buffering capacity that raised concern for nitrate accumulation at high N rates. Similarly, Delevatti et al. (2019) observed that the CP concentration was considerably linearly correlated with the rising N application rates (P< 0.01), which were associated with the increase in the DM yield. At the same time, both ADF and NDF showed a linear decreasing pattern with the increase in the N input, which suggests that the higher the N supply, the higher the quality of forage. In soils with low levels of S or Ca, targeted fertilization can be one of the factors for better forage digestibility via unique physiological processes. Indicatively, S deficiency can be remedied by soil amendments or diets, thus increasing the efficiency of ruminal fermentation, which ultimately increases forage digestibility (McDowell, 2003). Similarly, Ca applications have been associated with the changes in plant cell wall structure, which may enhance the digestibility of fibers and increase the nutrient availability for ruminants (McDowell, 2003). Extensive research has been conducted, which has concentrated on N application in wheat production with the primary aim of optimizing the grain yield, while comparatively fewer studies have shown the integrated relationships between soil nutrient management and forage quality (Bailey et al., 2007). Existing evidence suggests that forage growth stages have often influenced more on overall forage quality, whereas N application strongly affects CP but shows limited or inconsistent responses on other nutritive indices under certain production environments (Bailey et al., 2007).

### 6.5 Temperature and soil moisture

Temperature is one of the environmental factors with direct effects on plant composition (cell-wall deposition, lignification, N status, and soluble carbohydrate) and indirect effects on phenology (transition of plant from vegetative to reproductive stage) (Van Soest, 1988). The high and low temperatures have been a significant cause of massive agricultural yield losses during the 21st century (Han et al., 2025; Mahdavi et al., 2022). Higher temperatures have been shown in a large meta-analysis of grass nutritive traits to consistently depress forage nutritional value, indicating that rising temperatures can reduce digestibility while increasing fiber-related limitations at the whole-system level (Lee et al., 2017). Forages produced in lower temperatures usually have high quality because the cell wall components have not lignified, so they are easily digestible. Conversely, high temperature grown forages favor synthesis of lignin and fiber and thus the reduction in the amount of CP and forage digestibility and nutritional value (Dumont et al., 2015). Wheat forage is typically utilized during winters, so the temperature effect is particularly relevant because when temperature is low the forage preserves high digestibility by slowing lignification and maintaining a canopy for longer periods, whereas a rapid spring warming can shorten the high-quality grazing window by accelerating stem elongation and maturity (Van Soest, 1988). It is also important to note that evaluating the effect of temperature on forage quality in field conditions is challenging because of its inherent interaction with plant growth stages and maturation throughout the growing season. Exposure to low temperatures also plays an important role in determining wheat forage quality because low temperatures often stimulate the accumulation of water-soluble carbohydrates (WSC) in vegetative tissues. In a controlled environment, Lorenzo et al. (2015) reported that cooler temperatures increased sugar accumulation and hemicellulose content in wheat plants. These changes caused minimal effect on *in vitro* DM digestibility but resulted in higher *in vitro* rumen gas production, particularly in the higher sugar-accumulating genotype, by shifting plants from low to high temperature conditions, it sharply reduced rumen gas production. This shows that the temperature effect, either high or low, on wheat forage quality is not limited to changes in CP and fiber fractions, but cooler temperatures can also promote the accumulation of fermentable sugars and soluble proteins, thereby increasing the rapid fermentable substrates available in the rumen. Under some grazing situations, such compositional shifts may contribute to an increased risk of bloat in rumens (Lorenzo et al., 2015).

The availability of soil moisture has a very positive effect on the forage quality primarily through its effects on leaf area development, growth rate, and the timing of senescence, which alter the proportions of leaves, stem, and senescent material in the forage. A climate-manipulation meta-analysis reported by Dumont et al. (2015) highlights that forage quality responses showed a wide range under contrasting water availability conditions, from drought to irrigation. Drought stress produced inconsistent effects and on average, it was associated with an approximate 5% reduction in NDF and about 10% increase in forage digestibility. However, these responses varied considerably among trials. In some cases, the reductions in NDF may be partially compensated by slightly increase in lignin, which indicates the impact of drought on forage quality is highly influenced by stress timing and intensity. Across many forage-producing regions, water scarcity represents a more prevalent constraint than excessive soil moisture and often acts as a major factor limiting biomass production. However, under drought conditions, forage quality may increase in some cases because reduced plant biomass accumulation may concentrate nutrients within plant tissues (Grant et al., 2014). Habte et al. (2022) evaluated the performance of the various genotypes of Napier grass for forage by analyzing the agro-morphological and quality traits in the presence of moisture stress (severe and moderate water stress during the dry season and rainfed conditions during the wet season). Their analysis showed significant variations between agro-morphological and forage quality traits across moisture regimes. It is important to note that extreme moisture stress conditions caused a higher reduction in plant performance and forage quality than the moderate moisture climate. Although drought stress has received more attention in forage production studies, waterlogging and excessive soil moisture are becoming increasingly important constraints in wheat due to shifts toward more variable rainfall patterns in some regions. Waterlogging induces hypoxic or anoxic conditions in the root zone that disrupt root metabolism and nutrient uptake, which leads to reductions in leaf N content, photosynthetic capacity, and induce early chlorosis and senescence. Collectively, this may limit biomass accumulation and may also diminish the quality of the remaining green plant tissues (Pais et al., 2022).

### 6.6 Biotic stresses

Foliar plant pathogens (rusts, Septoria, mildew, etc.) and root diseases (e.g., *Rhizoctonia*, *Fusarium*) reduce the wheat forage quality by stressing the plant and destroying leaf tissue. These stresses can cause physiological changes, such as reductions in the leaf-to-stem ratio, CP content, and *in vitro* DM digestibility, and increases in fiber fractions (Anderson, 2015; Beckman and Whitney, 2025). Leaf biomass is usually more nutritionally valuable than the stem biomass among grass species (Mganga et al., 2021). Rust infection substantially destroys the green biomass and accelerates the leaf senescence, thereby hampering photosynthetic efficiency. Disease infection may reduce forage digestibility indirectly by reducing green leaf area, accelerating senescence, and increasing the proportion of fibrous or dead tissue in the harvested forage (Anderson, 2015). Beckman and Whitney (2025) reported that severe rust infection in corn and sorghum residues increases NDF by more than 10% while simultaneously reducing digestibility by more than 10%. Another example in pearl millet by Wilson et al. (1991), where they found that the forage digestibility decreased with the severity of rust infection, and the highest decreases were recorded in the early infection phases. Literature on the influence of insect herbivory on wheat forage quality has received comparatively less attention than yield reductions. However, insect herbivory normally causes a decrease in vegetative mass; they vary in their impact on the forage quality, and most of the time, the effect depends on the circumstances (Buxton and Fales, 1994). Most insects feed on leaves that target the mesophyll part, which are highly digestible, protein-rich, and essential for photosynthesis, which indirectly results in a lower leaf to stem ratio and reducing overall protein in the plants. For instance, aphid‐damaged plants show significantly reduced quality and amino nitrogen in leaves (Khattab, 2007). Grasses typically experience fewer insect-related constraints compared to legumes. Lenssen et al. (1991) reported that, under disease-free conditions, anthracnose-susceptible and resistant alfalfa (Saranac) did not differ markedly in overall forage quality, apart from stem NDF. However, when infected, the susceptible plants displayed slower shoot development, reduced CP content and stem digestibility. Additionally, leaf tissue quality was generally lower in infected susceptible plants, underscoring that disease-induced morphological and physiological changes can differently affect forage quality traits. In wheat forage systems, disease and pest management should not focus only on yield protection. It should also help maintain green leaf area, delay premature senescence, support regrowth after grazing or cutting, and preserve forage quality before grazing or harvest (USDA-NRCS, 2023; Simón et al., 2020).

## 7 Advancing wheat forage quality in wheat breeding: from conventional screening to predictive pipelines

### 7.1 Wheat forage quality targets and measurement frameworks

For breeding and management purposes, wheat forage quality can be defined by its ability of forage to support animal productivity and performance, i.e., maintenance, intake, milk yield, and growth rather than by any single quality trait. For breeding, the broad concept of forage quality must be translated into quantifiable phenotypic traits that can be measured accurately, consistently, and at the throughput required for effective selection and genomic prediction (Cobb et al., 2013; Furbank and Tester, 2011; Araus and Cairns, 2014).However, practical forage evaluation decisions typically depend on a set of chemical composition and digestibility indicators that are consistently associated with forage intake potential and energy supply (Zhang and Rocateli, 2017). Wheat forage refers to several types of plant biomass utilized for livestock feeding, including vegetative biomass grazed or clipped during late fall and early spring, whole-crop harvested for conserved forage (hay or silage), and wheat straw used as a ruminant feed resource particularly in regions where crop-livestock plays an important role (Kim et al., 2016). Forage that is grazed generally exhibits higher quality than conserved forage, particularly due to the selective grazing behavior of livestock and the higher concentrations of digestible nutrients typically found in fresh forage (Hancock et al., 2014). One important aspect of strengthening the concept of wheat forage quality is clearly distinguishing between primary measurements and derived indices. A routine forage analysis generally measures NDF, ADF, and N (represented as CP) (Zhang and Rocateli, 2017). Consistency in detergent-fiber terminology and analytical procedures is essential for reliable comparisons across forage literature. Variability in reporting methods, such as the use of ash correction and amylase treatment has often led to inconsistencies in NDF and ADF values. To address this, the animal nutrition community has proposed standardized analytical procedures, including the use of corrected fiber measures such as ash-free, amylase-treated NDF called as aNDFom, to improve reproducibility and comparability among laboratories and studies (Udén et al., 2005). In addition to structural carbohydrates, non-structural carbohydrates such as sugars and starch are rapidly digested by rumen microbes and play an important role in silage production. During silage fermentation, WSC supply substrate for lactic acid bacteria and pH decline, which helps stabilize fermentation (Castillo and Davidson, 2024). However, these measurements are relatively expensive and labor-intensive, which limits their application in large-scale breeding programs (Joshi et al., 2019). Therefore, the trait architecture matters for breeding because selection targets differ by production objective, such as for grazed fresh forage, breeders and producers often prioritize higher CP and digestibility during vegetative stages and early growth stage, while balancing both regrowth capacity and grazing tolerance (Xie et al., 2012). For whole-crop wheat silage, key targets include appropriate DM concentration at harvest, adequate WSC for fermentation, starch accumulation, low-to-moderate fiber concentration, digestibility, and favorable fermentation characteristics (Filya, 2003). Recommended DM concentration at ensiling depends on the storage system, with approximate targets of 30–35% DM for bunker or trench silos, 35–45% DM for conventional upright silos, and 35–50% DM for gas-tight upright silos (Belyea et al., 1993). In a dual-purpose system, breeders must also protect subsequent grain yield potential under grazing or cutting management, which underscores the importance of genotype × management interactions and multi-environment evaluation (West et al., 2024).

### 7.2 From reference chemistry to field-ready phenomics for wheat forage quality phenotyping

Across spectral phenotyping methods, predictions are only as reliable as the calibration behind them. Models perform best when the reference samples represent the range of materials being tested, including differences in genotype, maturity stage, moisture, environment, and management, and when accuracy is confirmed with independent validation data (Shenk and Westerhaus, 1994; Cherney et al., 2021; Geipel et al., 2021).

#### 7.2.1 Conventional reference phenotyping - wet chemistry

Conventional reference laboratory phenotyping based on wet chemistry analyses remains indispensable in forage quality breeding because it provides the benchmark phenotypes that are required for the calibration of rapid analytical techniques and the development of predictive modeling frameworks. Wheat forage quality phenotyping can be organized as a stepwise pipeline: wet chemistry provides the reference data, laboratory NIRS increases the number of samples that can be screened, portable NIR brings testing closer to the field, and canopy- or UAV-based spectral sensing offers a path toward non-destructive, plot-scale prediction (Andrés-Camelo et al., 2025; Cherney et al., 2021; Wijesingha et al., 2020; Araus and Cairns, 2014). In particular, wet-chemistry determinations serve as the reference standards for calibrating high-throughput platforms such as NIRS and for generating training datasets used in GS. In spectroscopy-based forage analysis, the predictive accuracy of NIRS models is directly dependent on the availability and quality of wet chemistry reference values used during calibration (Harris et al., 2018). Wet chemistry analysis typically quantifies key nutritive attributes such as NDF, ADF, CP, and digestibility. However, these methods are labor-intensive and often costly, as they require specialized laboratory setup, chemical reagents, safety management procedures, and waste disposal protocols. Large forage datasets used to develop chemometric prediction models illustrate this dependence on wet-chemistry assays, where conventional laboratory measurements are paired with spectral data to generate robust prediction models (Andrés-Camelo et al., 2025). However, the extensive sample preparation, which includes destructive sampling, drying, grinding, and subsequent laboratory analysis, makes conventional phenotyping inherently slow when applied at the breeding scale. As a result, laboratory throughput frequently becomes the primary bottleneck in large breeding and evaluation trials. This limitation has been highlighted in a study of wheat forage quality GS, where the phenotyping workload has been identified as a key factor restricting selection intensity and prolonging breeding cycles (Maulana et al., 2019a).

#### 7.2.2 Laboratory near-infrared reflectance spectroscopy

NIRS remains the main scalability of forage quality phenotyping; however, developing reliable calibration design remains a real bottleneck. It is widely adopted in forage evaluation because it can substantially reduce reliance on labor-intensive chemical analyses and digestibility assays once calibrations are properly developed. Despite this, it has practical constraints that matter to breeders. For instance, Yang et al. (2024) compared NIRS predictions obtained from dried and undried (wet) samples and found that dried samples generally provided higher prediction accuracy. They also found that prediction error increased with increasing sample moisture, and under very high moisture, water absorption bands can mask spectral signals associated with nutrients. A recent study by Castillo et al. (2025) indicated that predictive accuracy should not be assumed without proper validation. Variability among instruments, calibration datasets, and sample populations can influence model performance, highlighting the need for standardized validation and reporting practices for NIRS-based predictions.

#### 7.2.3 Portable and handheld near-infrared reflectance spectroscopy approach

Interest in the application of reduced-size, handheld NIRS for on-farm and field-based analysis has increased rapidly in recent years (Beć et al., 2020). The portable handheld NIRS device offers the potential to move forage-quality evaluation closer to the field, thereby further reducing labor and time. Despite this growing interest, published studies specifically validating portable NIRS for assessing wheat forage quality remain limited. Nevertheless, integrating this portable NIR into wheat forage breeding programs could substantially improve phenotyping efficiency by rapid screening of large numbers of samples in field conditions. Yet the independent evaluations of handheld NIR devices have shown that calibrations can lack robustness across diverse sample types. For instance, calibration models developed under control conditions may perform poorly when applied to other real-world samples, e.g., mixed haylage, corn silage, or total mixed rations with variable moisture contents and particle sizes. This highlights the need and reinforcing the argument that calibrations must be continuously expanded and independently validated (Cherney et al., 2021). Portable NIR devices have been shown to perform more consistently when applied to preprocessed, particularly dried and ground forage samples, where variation caused by moisture is minimized (Berzaghi et al., 2021). Moisture has been identified as a major source of prediction error in field-based NIR devices as water strongly absorbs NIR radiation and can mask signals associated with key forage quality traits (Yang et al., 2024). Despite these limitations, a recent study using a handheld NIRS device has demonstrated promising results for the rapid in-field prediction of forage quality traits such as CP, NDF, and ADF. These results indicate substantial potential for the use of portable NIR technologies in forage quality phenotyping after the development of crop-specific calibration models and robust external validation (Yamada et al., 2024).

#### 7.2.4 In-field spectral sensing and unmanned aerial vehicle hyperspectral imaging

To avoid reliance on conventional workflows, including sample collection-drying-grinding, increasing attention is being directed toward in-field prediction of fresh forage quality using spectral sensing combined with machine learning approach. For instance, Wright et al. (2025) evaluated an above-canopy spectral sensing system to estimate DM, ADF, NDF, and CP of fresh cool-season grass pastures throughout a grazing season using random forest regression. They explicitly quantified prediction errors and translated differences in predicted forage quality into expected differences in ME and protein supply within a beef-cattle nutrition model. At the canopy scale, unmanned aerial vehicles (UAVs) based hyperspectral imaging methods demonstrated promising capability for estimating forage yield and quality prediction in grassland systems. Wijesingha et al. (2020) reported that UAV-borne imaging spectroscopy could produce useful predictions for CP and ADF across diverse grassland environments, although model performance depended strongly on the diversity of calibration datasets and the regression methods employed. In a larger UAV hyperspectral study ~707 observations in grass-legume mixtures, multivariate regression using partial least squares regression generated reasonable validation errors for traits including DM yield, CP, DM digestibility, NDF, and indigestible NDF, whereas they found simple vegetation-index-based linear models were insufficient for accurate prediction. For wheat specifically, hyperspectral imaging has also been widely explored for monitoring N status and protein-related traits under field conditions. A review by Ma et al. (2022) highlighted the increasing use of machine learning to capture nonlinear relationships between spectral signatures and agronomic traits. Although these studies show that canopy and UAV-based spectral sensing can estimate forage quality traits, most of the evidence still comes from grassland or mixed-forage systems rather than wheat forage. For wheat breeding, these tools will need crop-specific calibration and independent validation before they can be used confidently for selection (Wijesingha et al., 2020; Geipel et al., 2021; Araus and Cairns, 2014). Nevertheless, future data collection and model refinement efforts will be necessary to determine the practical value of the spectral sensing systems for cost-effective, in-field monitoring of forage quality traits.

### 7.3 Genetic and genomic studies on wheat forage quality and straw nutritive traits

Research on wheat genetics and genomics is primarily concentrated within three application domains. The dual-purpose or forage wheat systems, in which plants are evaluated at vegetative or early reproductive stages for traits associated with forage nutritive value, including CP, NDF, ADF, ADL, SUG, and *in vitro* digestibility. Vegetative forage usually has higher CP, lower fiber, greater digestibility, and different tissue composition. Wheat straw as ruminant feed, its nutritional value is primarily evaluated using detergent fiber fractions (ADF, NDF, ADL) together with derived energy estimates such as ME. Wheat straw reflect cell wall concentration, lignification, and residual digestibility, which collectively determine its feeding value (Joshi et al., 2019). Biomass and bioenergy research, where straw or whole-plant biomass was evaluated for biorefinery settings, and the traits like saccharification potential, sugar release, and cell-wall recalcitrance serve as indirect measurements for structural and digestibility-related attributes (Malik et al., 2019). Although these research areas originated with different objectives, they increasingly rely on similar high-throughput phenotyping methods such as NIRS/visible near-infrared spectroscopy (VIS-NIR), enzymatic assays, and genome-wide analytical approaches, including single nucleotide polymorphism (SNP) arrays, genotyping-by-sequencing (GBS), whole-genome sequencing (WGS), GWAS, and GS. (**Table 3**) summarizes some of the GWAS studies carried out over the last decade for forage quality and related traits in wheat.

**Table 3:** Summary of genome-wide association studies for forage quality and related traits in wheat.

Reference	Genetics approach	Germplasm/population	Trait(s)	Genotyping	Number of MTAs/QTLs/QTNs	Chromosome(s)/ genomic regions
Li et al., 2015b	GWAS (MLM) + allele pyramiding (MAS concept)	Bread Wheat Winter Panel; 262 accessions	Stem WSC (internode-specific), link to TGW under drought	209 SSR markers	~33 MTAs	1B, 2B, 2D, 3A, 3B, 4B, 5A, 6B, 6D, etc.
Bellucci et al., 2015	GWAS	100 historical winter wheat (Scandinavia)	WSC; straw glucose and xylose release after pre-treatment; dual-purpose traits	DArT/SNP markers (marker IDs reported)	1 MTA	1B
Dong et al., 2016a	GWAS	166 Chinese bread wheat cultivars	Stem WSC	90K SNP array	23 QTLs/52 MTAs; 11 MTAs stable	All except 2A, 2D, 4D, 5B, 6A and 6D
Kaur et al., 2017	GWAS	288 diverse spring wheats	Culm cellulose content	DArT-seq/GBS; 21,073 SNPs	9 MTAs; 2 loci	1A, 1D, 2D, 3B, 4D, 5A, 5B, and 6B
Ovenden et al., 2017	GWAS (SNP vs DArT marker sets)	1314 tested; WSCC subset 312 lines + 46 varieties	WSCC (water-soluble carbohydrate concentration)	SNP + DArT arrays	8 MTAs	1A, 1B, 1D, 2D, and 4A
Joshi et al., 2019	GWAS	287 diverse spring wheat lines	Straw N, NDF, ADF, ADL, ash, ME	Unspecified	17 MTAs	1A, 2B, 3A, 5A and 5B
Fu et al., 2020	GWAS	166 diverse wheat accessions	Stem WSC	Unspecified	79 QTLs/62 stable	All 21 chromosomes
Guerra et al., 2021	GWAS	225 spring wheat	Stem WSC at anthesis	GBS (Illumina HiSeq 2000); 3243 SNPs retained	96 MTAs	1A, 3A, 4B, 5B, 5D, 6B, 6D, and 7B
Gaur et al., 2022	GWAS	301 bread wheat genotypes	Peduncle glucose/fructose/sucrose/fructans total NSC	35K Axiom; 14,571 SNPs	570 associations; 163 stable	All chr except 4D
Esposito et al., 2022	GWAS	Tetraploid wheat panel; 185 accessions	Straw composition traits incl. cellulose, NDF, ADF, ADL	Unspecified	20 QTNs	1B, 2A, 2B, 3A, 4A, 4B, 5A, 6B, 7A, 7B
Li et al., 2024	VIS/NIR prediction + GWAS	326 wheat accessions	Cellulose crystallinity index (CrI); links to NDF/ADF by haplotype	WGS-derived SNPs; 2.09M	14 significant MTAs; 13 QTL	1A, 2A, 2B, 2D, 3B, 3D, 4B, 5B, 6A
Dong et al., 2025	GWAS	166 wheat accessions	Stem lignin, cellulose, hemicellulose	660K SNP chip	1146 significant MTAs	Across all three subgenome

#### 7.3.1 Genome-wide association study

A small number of wheat GWAS have explicitly evaluated ruminant-relevant forage and straw quality traits (**Table 3**). A larger set of genomic research has focused on stem carbohydrate reserves, particularly WSC and fructans (polymers of fructose). These carbohydrate traits are biologically relevant to the energy value of cereal biomass for ruminant nutrition and directly contribute to forage energy and palatability (Van Soest, 1994; Fulkerson and Donaghy, 2001). However, these traits are commonly investigated within the context of plant physiological processes associated with drought adaptation and grain-filling dynamics rather than forage nutritive quality per se (**Table 3**). For instance, GWAS by Li et al. (2015b) identified genomic regions for stem WSC linked to TGW under drought (**Table 3**). Bellucci et al. (2015) conducted a GWAS using a panel of 100 wheat varieties, in which 93 were genotyped and used for GWAS. Lignocellulosic straw (glucose, xylose), glucose released after enzymatic hydrolysis of biomass, and other yield-related traits were evaluated. A total of 6 QTLs were identified, of which only one QTL was associated with glucose released after enzymatic hydrolysis (**Table 3**). Similarly, Dong et al. (2016a) performed a GWAS using a panel of 166 Chinese bread wheat cultivars to investigate the genetic basis of WSC. They identified 52 significant marker-trait associations (MTAs), which were consolidated into 23 putative QTLs (**Table 3**).

Kaur et al. (2017) conducted a GWAS using 288 genetically diverse spring wheat genotypes to investigate the genetic architecture of culm cellulose content, where they identified 9 genomic regions significantly associated with variation in cellulose content (**Table 3**). They further reported that these loci may be directly or indirectly involved in the regulation of cellulose biosynthesis in wheat culms, highlighting the potential for genetic improvement of cellulose content in bread wheat and other cereals with implications for enhancing culm strength as well as improving biofuel production. Joshi et al. (2019) evaluated a population of 287 diverse spring wheat lines for straw fodder quality traits, including ADF, NDF, ADL, ash, and ME, alongside grain and straw yield association. They detected associations distributed across chromosomes in the A and B sub-genomes, with a region on chromosome 2B associated with ADF and ADL, and ME on chromosome 5B (**Table 3**). This is one of the most directly forage-relevant wheat association studies because it targets straw nutritive value traits widely used by animal nutritionists.

Ovenden et al. (2017) conducted a GWAS to investigate WSC concentration using multi-environmental data collected under contrasting water availability. Eight significant MTAs were detected on chromosomes 1A, 1B, 1D, 2D, and 4A (**Table 3**). They also demonstrated that WSC loci were largely independent of major maturity loci, suggesting genetic separation between carbohydrate accumulation and phenological development. For forage breeding, this is relevant because maturity strongly affects quality traits, but selection for carbohydrate accumulation may not need to rely entirely on changes in phenology. Another study by Fu et al. (2020) performed a GWAS to investigate the genetic relation of stem WSC content with grain yield in wheat and identified 62 stable loci, in which 36 were potentially new loci, in which 13 showed pleiotropic effects with thousand kernel weight (**Table 3**). Notably, they developed and validated five competitive allele-specific PCR (KASP) markers linked to favorable alleles to provide a direct pathway for marker-assisted selection (MAS).

Taken together, these studies show that genomic regions controlling WSC accumulation, cellulose content, lignocellulosic degradability, and straw fodder quality can be detected in wheat. However, the evidence base remains fragmented because many studies were designed for drought adaptation, grain filling, lodging resistance, or bioenergy rather than forage quality per se. For wheat forage breeding, the next step is to evaluate whether loci identified for WSC, cellulose, lignin-related traits, digestibility, and straw nutritive value are stable across forage harvest stages, grazing or cutting management, environments, and genetic backgrounds.

#### 7.3.2 QTL mapping and marker-assisted selection

Compared with direct genetic studies of wheat forage quality traits such as CP, NDF, ADF, ADL, lignin, and *in vitro* digestibility, biparental QTL mapping and functional marker development are much more developed for stem WSC, particularly fructan synthesis and remobilization. However, most WSC mapping studies in wheat were developed in the context of drought adaptation and grain filling rather than forage quality. Their relevance to forage breeding is therefore indirect and should be validated at forage-relevant harvest stages and management conditions. (**Table 4**) summarizes some of the QTL mapping studies carried out over the last decade for forage quality and related traits in wheat. A notable example is the work by Dong et al. (2016b) that provides one of the clearest examples of moving from candidate gene discovery to marker development for wheat stem WSC. They cloned wheat sucrose: sucrose 1-fructosyltransferase genes (TaSST) and mapped them to chromosomes 4A, 7A and 7D. Using a recombinant inbred lines (RILs) population, they identified three QTL associated with stem WSC and developed a gene-specific cleaved amplified polymorphic sequence (CAPS) marker for TaSST-D1 (**Table 4**). The gene identification, QTL localization, and functional marker development make this study particularly relevant for breeding applications, as it provides a platform for MAS.

**Table 4:** Summary of few representative linkage/QTL mapping studies for forage quality and related traits in wheat.

Reference	Genetics approach	Germplasm and size	Trait(s)	Genotyping	Number of MTAs and QTLs	Chromosome(s)/genomic regions
Yang et al., 2007	Linkage QTL + epistasis + QTL×E	Bread wheat DH	Stem WSC accumulation/remobilization under drought vs well-watered	Unspecified	~4 QTLs	2D and 3B
Rebetzke et al., 2008	Linkage QTL	Bread wheat; 3 mapping populations	Stem WSC and agronomic traits	Unspecified	~90 QTLs	Across all 21 chromosomes except 6A
Dong et al., 2016b	Linkage mapping and candidate gene analysis (TaSST gene cloning and functional marker development)	149 Chinese wheat cultivars; RIL population	Stem WSC	SNP markers and CAPS functional marker (WSC7D) developed from TaSST-D1 polymorphism	3 QTLs	7DS, 4BS, and 7AS
Li et al., 2020	Linkage QTL + epistasis networks	120 bread wheat RIL	Stem WSC-related traits	Unspecified	95 additive/88 epistatic QTLs	1A, 1B, 2A, 2B, 2D, 3B, 4A, 4B, 5A, 6A, 6B and 7A
Shafaei et al., 2025	Linkage QTL	167 F9 lines	Enzymatic digestibility of straw (sugar release after cellulase/xylanase)	DArT; 662 markers	3 QTLs	7A and 6D

Earlier linkage mapping studies also revealed the complex genetic architecture underlying WSC accumulation in wheat. Yang et al. (2007) identified approximately 4 QTLs associated with stem WSC and remobilization under contrasting water conditions, highlighting strong genotype-by-environment (G × E) interactions in carbohydrate (**Table 4**). Similarly, Rebetzke et al. (2008) reported a large number of genomic regions, approximately 90, controlling WSC. Li et al. (2020) examined the genetic basis of stem WSC using a population of 120 RILs under both drought-stress and well-watered conditions across three environments. They identified 95 additive QTLs and 88 pairs of epistatic QTLs, including 13 additive QTLs that were consistently detected across two of the three water environments. These QTLs formed 19 clusters on chromosomes 1B, 2A, 2B, 2D, 3B, 4B, 5A, 6A, 6B, and 7A, suggesting possible genetic linkage or pleiotropy for stem WSC traits (**Table 4**).

#### 7.3.3 Genomic selection and genomic prediction studies

In GS, individuals are selected based on genome-wide marker profiles that collectively capture genetic variation underlying overall plant performance (Varshney et al., 2015). This capability underpins GS as an alternative to traditional MAS. Consequently, unlike MAS, GS does not rely on prior identification of major-effect QTLs (Guo et al., 2020). All available markers in GS are simultaneously fitted to a statistical model using a training population (TP) that has both phenotype and genotype data. Marker effects estimated from the TP are then used to compute genomic estimated breeding values (GEBVs) for selection of candidates that have been genotyped but not phenotyped (Hayes and Goddard, 2001). Selection decisions can thus be made directly on these GEBVs without additional phenotyping. Importantly, by using genome-wide markers, GS greatly increases the chance of capturing linkage disequilibrium with the QTL underlying a trait (Goddard and Hayes, 2007). Recent reviews emphasize that GS tends to increase genetic gain, reduce the need for extensive phenotyping, and accelerate cultivar development by shortening the breeding cycle (Wróbel et al., 2025). In forage breeding, GS is only beginning to be explored: for example, GS has been tested for quality traits in alfalfa (Annicchiarico et al., 2021; Biazzi et al., 2017), and particularly in wheat for forage quality e.g. forage quality GS in winter wheat by Maulana et al. (2019a) and for stem WSC content in wheat by Ovenden et al. (2018) demonstrating its potential utility. (**Table 5**) provides a summary of the GS studies conducted over the past decade for forage quality and related traits in wheat.

**Table 5:** Summary of few representative genomic-selection studies for forage quality and related traits in wheat.

Reference	Genetics approach	Germplasm and size	Trait(s)	Genotyping	Prediction accuracy	GS models
Maulana et al., 2019a	GS	298 winter wheat genotypes	CP, ADF, NDF, SUG, and lignin	90K SNP array	0.34 to 0.61	RRBLUP, Gaussian kernel, Bayesian LASSO
Ovenden et al., 2018	GS	358 wheat genotypes	Stem WSC concentration	9k iSelect array, 4883 SNPs scored, 4,162 retained	0.20 to 0.64	Factor-analytic mixed model genomic prediction

Maulana et al. (2019a) evaluated GS for forage quality traits using a hard red winter wheat diversity panel comprising 298 genotypes, which was genotyped with a 90K SNP array and phenotyped for CP, ADF, NDF, SUG, and lignin. Genomic prediction was assessed using three models, such as ridge regression best linear unbiased prediction (RRBLUP), Bayesian LASSO (BL, where LASSO: least absolute shrinkage and selection operator), and Gaussian kernel (GAUSS) to estimate breeding values for forage quality traits. Prediction accuracies across traits ranged from 0.34 to 0.61 and were generally comparable among the models tested. They also examined the impact of TP size and marker density on prediction accuracy and found that increasing TP size showed more improved accuracy than increasing marker density. An optimal TP of approximately 150 lines was suggested, at which most traits except SUG achieved more than 90% of the accuracy obtained with a TP size of 250. Additionally, CP reached its highest accuracy with a relatively small number of only 1000 markers required (**Table 5**).

GS remains an evolving and exciting approach, and ongoing development of statistical and computational models continues to improve the accuracy of GEBV prediction. Several GS models have been developed that incorporate G×E interactions, thereby enhancing prediction accuracy across diverse environments. Ovenden et al. (2018) evaluated 358 wheat lines, selected to minimize variation in maturity under rainfed and irrigated conditions across two locations and two years to predict WSC concentration. Genotyping was performed using the Illumina 9k iSelect array, yielding 4,883 SNPs and 4,162 markers after filtering. Whole-genome marker profiles were analyzed using factor analytic mixed models to generate GEBVs for individual environments and for cluster environments. They reported that additive genetic variance for WSC was smaller than non-additive genetic variance and G × E interaction effects, indicating that genotypic values were influenced more by residual genetic effects than by additive breeding values. As a result, GEBVs were considered highly suitable for parent selection in crossing blocks, though less reliable for predicting the absolute phenotypic performance ranking of specific lines across unpredictable environments. They noted that GS can increase genetic gain per unit time relative to phenotypic selection if breeding cycle length is reduced by 50% and emphasized that reliable prediction requires training environments that are representative of target production environments (**Table 5**).

## 8 Challenges and future directions for improving wheat forage quality

Despite the recent advances in wheat forage quality genomics, several important knowledge gaps remain in the genetic dissection and predictive improvement of wheat forage quality. It is important to fill these gaps for developing robust predictive breeding pipelines that integrate high-throughput phenotyping and GS. First, major forage quality traits remain underrepresented in wheat genetics research relative to traits related to stem carbohydrate reserves or bioenergy-oriented straw composition. Much of the existing literature has focused on WSC and fructan fractions because of their direct relevance to grain filling and drought resilience (Yang et al., 2007; Li et al., 2015b; Fu et al., 2020; Guerra et al., 2021). However, only a small number of wheat genomic studies directly target the major forage quality traits such as, CP, NDF, ADF, ADL, SUG, and *in vitro* digestibility (Joshi et al., 2019; Esposito et al., 2022). Currently, GS represents the most clearly deployable framework for breeding improvement of these complex forage quality traits (Maulana et al., 2019a, b). This highlights an important research opportunity for future studies to prioritize multi-location GWAS and genomic prediction models based on forage harvesting/clipping growth stage rather than relying on extrapolations from maturity-stage straw composition.

Second, phenotypic standardization remains a major bottleneck for cross-study comparability and predictive modeling. Forage quality traits are quantified using different operational definitions and analytical methods. For instance, fiber and digestibility may be represented by detergent fiber fractions (NDF, ADF, ADL), cellulose crystallinity, sugar-release assays, or WSC, each reflecting different aspects of the same feed-value concept (Maulana et al., 2019a, b). To improve predictive modeling frameworks, breeding programs would benefit from greater methodological harmonization, including standardized trait definitions, reporting units, and laboratory protocols, such as consistent use of aNDFom vs NDF, clearly defined lignin determination methods, and standardized *in vitro* digestibility incubation periods (48 h). In addition, the development of robust cross-laboratory spectroscopy calibration that remains stable across environments and germplasm is critical, similar in scope to the integrated VIS-NIR to GWAS pipeline recently implemented for the analysis of cellulose crystallinity (Li et al., 2024).

Third, validation and practical deployment of genomic identifications remain inconsistent across studies. Although numerous GWAS, QTL, and GS have identified genomic regions associated with WSC, straw fiber composition, and digestibility, relatively few studies have advanced these results toward applied breeding tools. A particular example is the tetraploid wheat straw quality study in which mixed linear models-GWAS identified candidate markers subsequently validated using high-resolution melting and RNase H2-dependent PCR assays and explicitly proposed for MAS (Esposito et al., 2022). Similarly, GWAS focuses on WSC by Dong et al. (2016b) report loci with stability across multiple environments; however, validation in independent populations or demonstration of practical deployment within breeding programs remains relatively limited. Future studies should therefore prioritize cross-population validation of identified genomic regions, preferably using different germplasm, and the development of breeder-friendly marker such as KASP assays. In addition, selection responses should be evaluated under realistic breeding constraints, similar to genomic prediction study demonstrating that training populations derived from related environments substantially improve predictive accuracy (Ovenden et al., 2018).

Fourth, genotype x management interactions remain poorly characterized in dual-purpose wheat production systems. A multi-location study by Joshi et al. (2019) found strong year and location effects on all wheat straw fodder traits, while genome-wide analyses of WSC concentration have demonstrated that certain loci exhibit environment-specific expression, particularly under drought conditions (Ovenden et al., 2017). This shows the importance of adopting multi-environment and multi-management experimental designs into predictive breeding pipelines, explicitly incorporating key agronomic variables such as harvest/clipping stage, N management, and water availability as covariates or interaction terms within GWAS and GS models.

Finally, progress has been made in identifying genomic regions, but functional interpretation of these loci remains incomplete. Recent studies have begun to move beyond simple lists of MTAs by integrating QTL clustering, haplotype analyses, tissue-specific expression filtering, and candidate gene prioritization such as, GWAS analyses of cellulose crystallinity and enzymatic digestibility traits that combine genomic association signals with transcriptomic information to refine candidate gene lists (Li et al., 2024). The next step of research should focus on systematic functional validation of candidate loci, including characterization of allelic variants, regulatory polymorphisms, and gene expression patterns. Where feasible, gene-editing approaches or targeted mutagenesis could further clarify causal relationships. Addressing these challenges will be fundamental for advancing wheat forage quality genomics beyond largely descriptive studies toward predictive and deployment-oriented breeding strategies aimed at accelerating genetic improvement in dual-purpose wheat systems.

## 9 Conclusion

Wheat forage stands out as a potent nutritive resource in cool-season grazing systems. Young, vegetative wheat provides high protein, energy, and palatability that enables higher livestock performance, especially when warm-season forages are unavailable. However, this review underscores that forage value is dynamic, quality declines rapidly with maturity due to fiber accumulation and carbohydrate partitioning (strongly influenced by wheat growth stage, temperature, water, and nutrient management etc.). We emphasize the need to view quality functionally, not just chemical composition, but also intake, digestion, and animal response. Key management insights include limiting grazing to early growth or using multiple cuttings, supplementing minerals (especially Ca), and monitoring nitrates or bloat under stress conditions. These practices can harness wheat’s nutritive potential while reducing risks. Genetically, the review highlights that modern genomic tools are only beginning to be applied to wheat forage quality traits. Encouragingly, GWAS has identified loci for stem WSC (fructans) and fiber composition (NDF, ADF, ADL etc.), and GS models show promise for predicting complex quality traits (CP, NDF, digestibility etc.) in diverse germplasm. Currently, many traits remain understudied, and calibration of high-throughput phenotyping (e.g. NIRS) is needed for breeding. In closing, maintaining wheat pastures as high-quality forage will rely on integrated approaches, such as agronomic practices tailored to maximize vegetative growth, nutritional programs to balance diets, and breeding strategies guided by both field evaluation and genomic prediction. By clearly defining forage quality targets (high digestibility, stable yield, safe metabolic profile) and leveraging modern tools, researchers and producers can enhance dual-purpose wheat systems for robust winter feed production and animal gains.
